# Diallyl disulfide induces DNA damage and growth inhibition in colorectal cancer cells by promoting POU2F1 ubiquitination

**DOI:** 10.7150/ijbs.91206

**Published:** 2024-01-21

**Authors:** Longzheng Xia, Jinguan Lin, Mingjing Peng, Xianjie Jiang, Qiu Peng, Shiwen Cui, Wenlong Zhang, Shizhen Li, Jiewen Wang, Linda Oyang, Shiming Tan, Zifan Hu, Nayiyuan Wu, Yanyan Tang, Xia Luo, Zongyao Ren, Yingrui Shi, Qianjin Liao, Yujuan Zhou

**Affiliations:** 1 Hunan Key Laboratory of Cancer Metabolism, Hunan Cancer Hospital and the Affiliated Cancer Hospital of Xiangya School of Medicine, Central South University, Changsha, 410013, Hunan, China.; 2 Hengyang Medical School, University of South China, Hengyang, 421001, Hunan, China.; 3 Public Service Platform of Tumor organoids Technology, 283 Tongzipo Road, Changsha, 410013, Hunan, China.

**Keywords:** diallyl disulfide, the pentose phosphate pathway, DNA damage, apoptosis, POU2F1 ubiquitination

## Abstract

Previous studies have demonstrated that diallyl disulfide (DADS) exhibits potent anti-tumor activity. However, the pharmacological actions of DADS in inhibiting the growth of colorectal cancer (CRC) cells have not been clarified. Herein, we show that DADS treatment impairs the activation of the pentose phosphate pathway (PPP) to decrease PRPP (5-phosphate ribose-1-pyrophosphate) production, enhancing DNA damage and cell apoptosis, and inhibiting the growth of CRC cells. Mechanistically, DADS treatment promoted POU2F1 K48-linked ubiquitination and degradation by attenuating the PI3K/AKT signaling to up-regulate TRIM21 expression in CRC cells. Evidently, TRIM21 interacted with POU2F1, and induced the K272 ubiquitination of POU2F1. The effects of DADS on the enhanced K272 ubiquitination of POU2F1, the PPP flux, PRPP production, DNA damage and cell apoptosis as well as the growth of CRC tumors* in vivo* were significantly mitigated by TRIM21 silencing or activating the PI3K signaling in CRC cells. Conversely, the effects of DADS were enhanced by TRIM21 over-expression or inhibiting the PI3K/AKT signaling in CRC cells. Collectively, our findings reveal a novel mechanism by which DADS suppresses the growth of CRC by promoting POU2F1 ubiquitination, and may aid in design of novel therapeutic intervention of CRC.

## Introduction

Colorectal cancer (CRC) is the third most common cancer and one of the leading causes of cancer-related death in both men and women in industrialized countries 1. Currently, therapeutic strategies for the intervention of CRC include surgical resection of tumors, adjuvant chemotherapies, radiotherapy and targeted therapies as well as immunotherapies. Unfortunately, the therapeutic efficacy of these therapeutic strategies remains limited, particularly for those advanced stages of CRC. Indeed, the rapid progression and the development of chemoresistance are the main causes for CRC-associated mortality 2, 3. Hence, discovery of new effective and safe therapies and understand their pharmacological actions are of significance in the management of CRC patients. Conceivably, understanding the pathogenesis of CRC is crucial for identifying the key molecular driver events associated with CRC tumorigenesis and progression, and for uncovering the potential targets for the development of new therapeutic strategies.

It is widely recognized that tumor cells prefer to generate energy through aerobic glycolysis or the Warburg effect to support their aggressive malignancy 4-6. Aerobic glycolysis can breach into the pentose phosphate pathway (PPP) to generate ribulose-5-phosphate and NADPH for DNA and fatty acid synthesis. The PPP flux is positively regulated by POU2F1 expression 7. Hence, therapeutic targeting the aerobic glycolysis and the PPP regulatory network may be valuable for the control of tumor cell growth 6. Currently, several small molecules targeting cancer metabolism have been developed for potential intervention of cancers, including hexokinase inhibitors, fatty acid metabolism inhibitors 8-10. However, the therapeutic efficacy of these inhibitors has not been demonstrated in CRC. Accordingly, uncovering new therapeutic agents targeting the metabolic network will be urgently needed.

Diallyl disulfide (DADS), an accessible garlic extract, has been used for fighting bacterial or fungal infection, cardiovascular and cerebrovascular diseases, and cancers 11. Our previous study demonstrated that DADS treatment significantly inhibited the proliferation metastasis of gastric and leukemia cells 12, 13. In addition, DADS treatment suppresses the malignant behaviors of CRC cells by attenuating the Rac1-Pak1-LIMK1-Cofilins signaling pathway 14. Hence, DADS is a potential therapeutic agent for CRC. More importantly, other studies have reported that DADS suppresses cell stemness, proliferation, metastasis, glucose metabolism, and drug resistance in other types of malignant tumors 15-17, suggesting that DADS may be an attractive therapeutic strategy for different types of malignant tumors. However, the precise mechanisms that underlie the pharmacological actions of DADS in inhibiting cancer metabolism have not been clarified.

The current study employes* in vitro* human CRC cells and* in vivo* xenograft CRC tumor to investigate the pharmacological mechanisms by which DADS inhibited the growth of CRC. Our findings indicated that DADS significantly suppressed the growth, glycolysis and the PPP flux in CRC cells in a POU2F1-dependent manner. Mechanistically, DADS enhanced POU2F1 ubiquitination and degradation by attenuating the PI3K/AKT signaling to up-regulate TRIM21 expression, leading to an enhanced TRIM21-mediated POU2F1 K48-linked ubiquitination and inhibition of the PPP flux in CRC cells. Therefore, our findings may offer new insights into the pharmacological mechanisms that underlie the actions of DADS in inhibiting the growth of CRC and aid in design of new therapies for CRC by targeting the PI3K/AKT/TRIM21/POU2F1 axis.

## Results

### DADS inhibits the growth of CRC cells *in vivo* and *in vitro*

**Figure 1:**
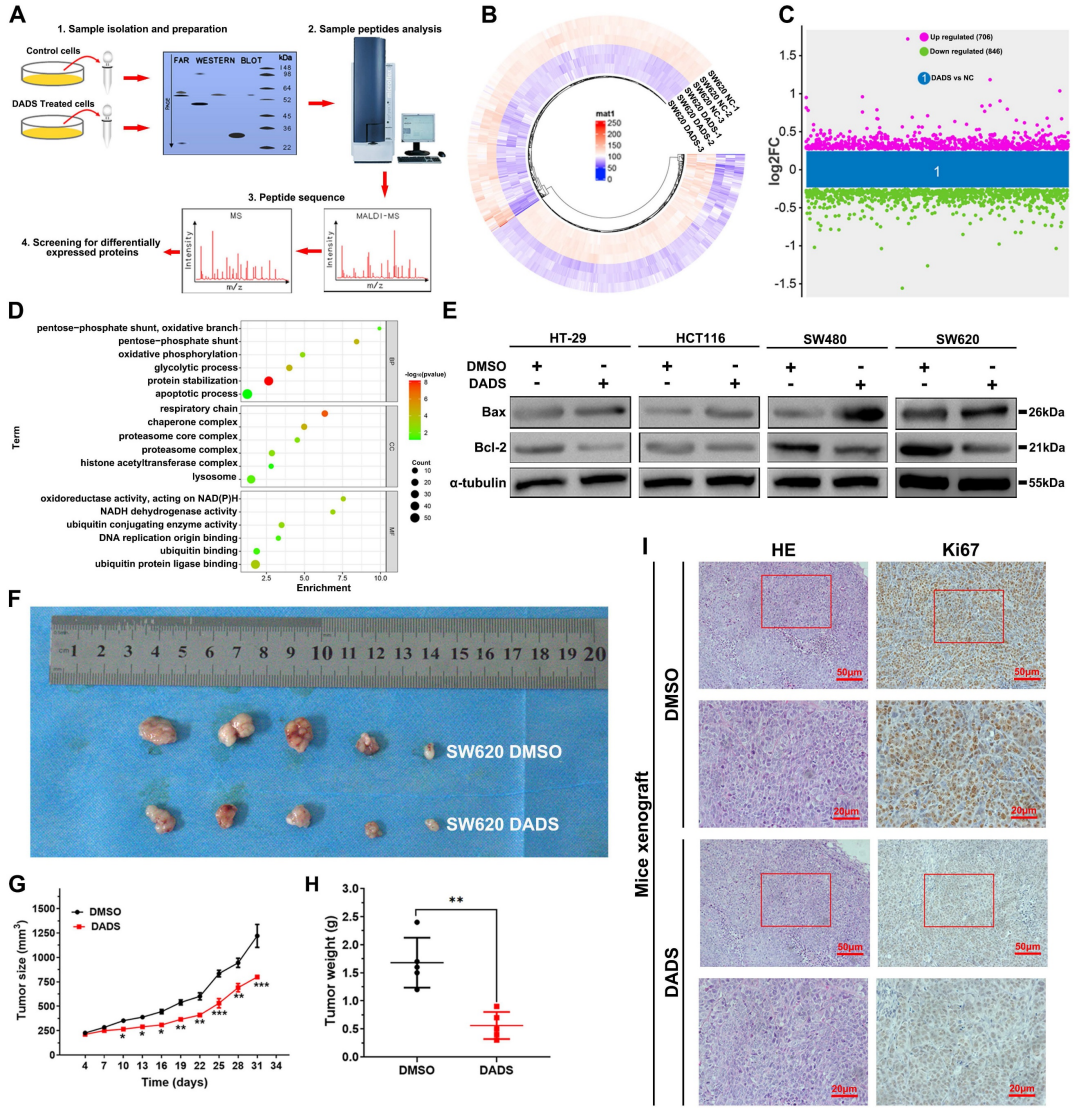
** DADS inhibits the growth of CRC cells *in vitro*.** (**A**) A schematic diagram to illustrate the proteomic analysis of vehicle-treated and DADS-treated SW620 cells. (**B**) Heatmap analysis of DEPs in the DADS-treated SW620 cells. (**C**) The distribution of DEPs in the DADS-treated SW620 cells. The DEPs were determined, based on the criteria of absolute fold change ≥1.2 and P-value <0.05. (**D**) The GO enrichment analysis of the potential functions of 1552 DEPs. (**E**) Western blot analysis of Bax and Bcl-2 expression in the indicated cells, which were treated with vehicle DMSO or DADS at 45 μg/mL (SW620), 42 μg/mL (SW480), 32 μg/mL (HCT116) or 30 μg/mL (HT-29) for 48 h. (**F-H**) Individual BALB/c nude mice were injected subcutaneously with 5×10^6^ SW620 cells indicated. When the tumors reached at 200 mm^3^, the mice were injected intraperitoneally with normal saline or DADS (100 mg/kg) every other day up to 4 weeks. The dynamic growth of tumors was measured longitudinally (n=5 per group). At the end of observation, their tumors were dissected, photoimaged, and weighed. (**I**) H&E staining and immunohistochemistry images confirmed Ki67 expression (magnification x 200, scale bars 50 μm, magnification x 400, scale bars 20 μm). Data are representative images or expressed as the mean ± SD of each group of samples from three separate experiments. *P<0.05, **P<0.01, ***P<0.001.

Our previous studies have demonstrated that treatment with DADS inhibits the metastasis of CRC cells by suppressing Rac1-mediated EMT (epithelial mesenchymal transition). 13, 14. In this study, we initially tested the impact of DADS treatment on the proliferation of CRC cells. We observed that DADS treatment significantly suppressed the proliferation and clonogenicity of human CRC HT-29, HCT116, SW480, and SW620 cells *in vitro* (P<0.05, **Figure S1A-B**). To gain insights into the biological actions of DADS in CRC cells, we conducted a label-free quantitative proteomic analysis of vehicle-treated and DADS-treated SW620 cells (**Figure 1A**) and identified 1552 differentially expressed proteins (DEPs), including 706 up-regulated DEPs and 846 down-regulated DEPs in the DADS-treated cells, relative to the vehicle-treated control SW620 cells, based on the criteria of an absolute fold change ≥1.2 and P<0.05 (**Figure 1B-C**). The gene ontology (GO) enrichment analysis exhibited that these DEPs were highly relevant to the biological processes of apoptosis, glycolysis, and protein stabilization (**Figure 1D**), suggesting that DADS might regulate the apoptosis of CRC cells. To address it, we performed Western blot and TUNEL assay to detect the effect of DADS on CRC cell apoptosis, and observed that DADS treatment significantly increased the frequency of TUNEL apoptotic CRC cells and upregulating Bax expression, compared to that of vehicle-treated control cells (P<0.05, **Figure S1C-D and Figure 1D**). Additionally, DADS treatment significantly inhibited the growth of xenograft CRC tumors in nude mice by reducing the tumor volumes and weights, relative to that of mice receiving vehicle (P<0.05, **Figure 1F** and **Figure S1E**). Consistently, immunohistochemistry (IHC) displayed that compared with the control tumors, lower levels of Ki-67 and Bcl-2 expression, but higher levels of Bax and cleaved caspase 3 expression were detected in the tumors from the mice receiving DADS treatment (**Figure 1H and Figure S1F**). Hence, DADS treatment inhibited the growth of CRC cells by inducing their apoptosis *in vivo* and *in vitro*.

### DADS enhances DNA damage, contributing to the apoptosis of CRC cells

Next, we explored the pharmacological actions of DADS in inhibiting CRC growth and inducing their apoptosis. DNA damage and its related genomic instability are associated with triggering apoptosis of cells. Actually, induction of DNA damage triggers the apoptosis of several types of cancer cells 18-20.

**Figure 2:**
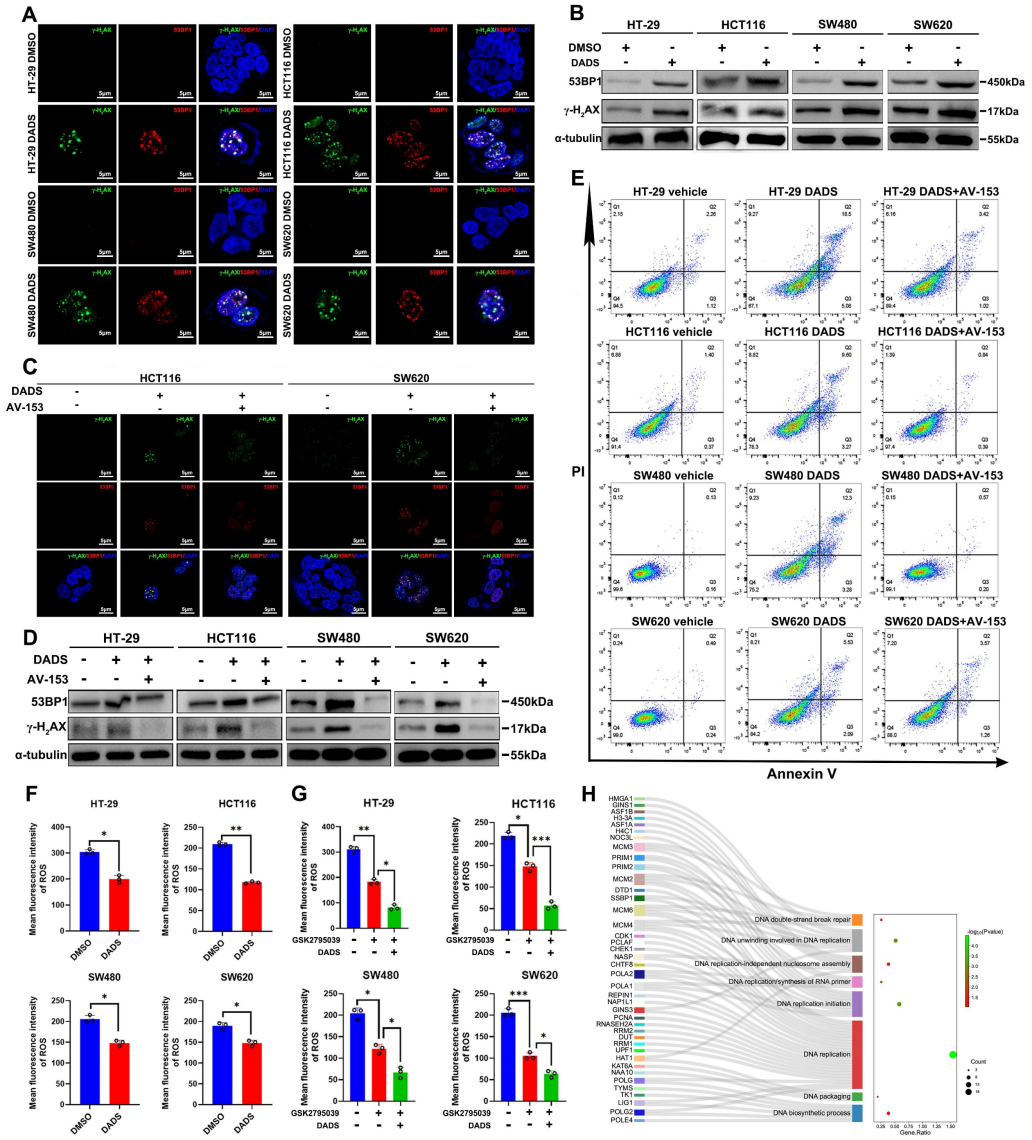
** DADS enhances DNA damage and apoptosis of CRC cells, independent of elevated ROS levels.** (**A-B**) Immunofluorescent and western blot analyses of γ-H_2_AX and 53BP1 expression in the indicated cells (magnification x 1000, scale bars 5 μm). (**C**) Immunofluorescent analysis of γ-H_2_AX and 53BP1 expression in the indicated cells following treatment with DADS alone or combination with 10 mM AV-153 (magnification x 1000, scale bars 5 μm). (**D**) Western blot analysis of γ-H_2_AX and 53BP1 expression in the indicated cells following treatment with DADS alone or combination with 10 mM AV-153. (**E**) Flow cytometry analysis of apoptotic cells. (**F-G**) The intracellular ROS levels were detected by flow cytometry in the indicated cells. (**H**) Sankey + dot plot analysis of DEPs. Data are representative images or expressed as the mean ± SD of each group of samples from three separate experiments. *P<0.05, **P<0.01, ***P<0.001.

Accordingly, we assessed whether DADS could modulate spontaneous DNA damage response in CRC cells by immunofluorescence. Notably, DADS treatment significantly increased the formation of γ-H_2_AX and 53BP1 (two markers of the early DNA damage) foci in CRC cells (P<0.05, **Figure 2A** and **Figure S2A**). A similar pattern of up-regulated γ-H_2_AX and 53BP1 expression was detected in CRC cells by Western blot assays (**Figure 2B**), suggesting that DADS treatment might enhance DNA damage and cause genomic instability. Additionally, DADS treatment significantly increased the percentages of apoptotic CRC cells and DNA damage, which was significantly mitigated or abrogated by treatment with AV-153 (an antimutagenic molecule) (P<0.05, **Figure 2C-E** and **Figure S2B-D**). More importantly, AV-153 reversed the inhibitory effect of DADS and enhanced the proliferation of CRC cells (P<0.05, **Figure S2E**). Hence, we hypothesized that DADS treatment might trigger apoptosis by enhancing DNA damage and genomic instability in CRC cells.

The imbalance between oxidation and antioxidant effects can lead to DNA damage in the body 21. Wherein, oxidative DNA damage, particularly induced by reactive oxygen species (ROS), is one of the most common types of DNA damage in cells 21, 22. Accordingly, we examined whether DADS treatment could increase intracellular ROS levels in CRC cells. Actually, DADS treatment significantly decreased the levels of intracellular ROS in CRC cells, relative to that of control cells (P<0.05, **Figure 2F**). Furthermore, DADS treatment enhanced the inhibitory effect of GSK2795039 (an inhibitor of NADPH oxidase 2) on ROS production in CRC cells (P<0.05, **Figure 2G**), indicating that DADS treatment did not promote ROS production to induce DNA damage in CRC cells. However, interestingly, the proteomic analysis revealed that DEPs were highly relevant to the biological processes of DNA double-strand break (DSB) repair, DNA replicate and DNA biosynthetic process (**Figure 2H**), suggesting that DADS might affect the DSB repair. Collectively, DADS treatment promoted the DNA damage-mediated apoptosis by regulating DNA damage repair, leading to inhibition of CRC growth.

### DADS suppresses the pentose phosphate pathway to cause DNA damage repair deficiency in CRC cells

**Figure 3:**
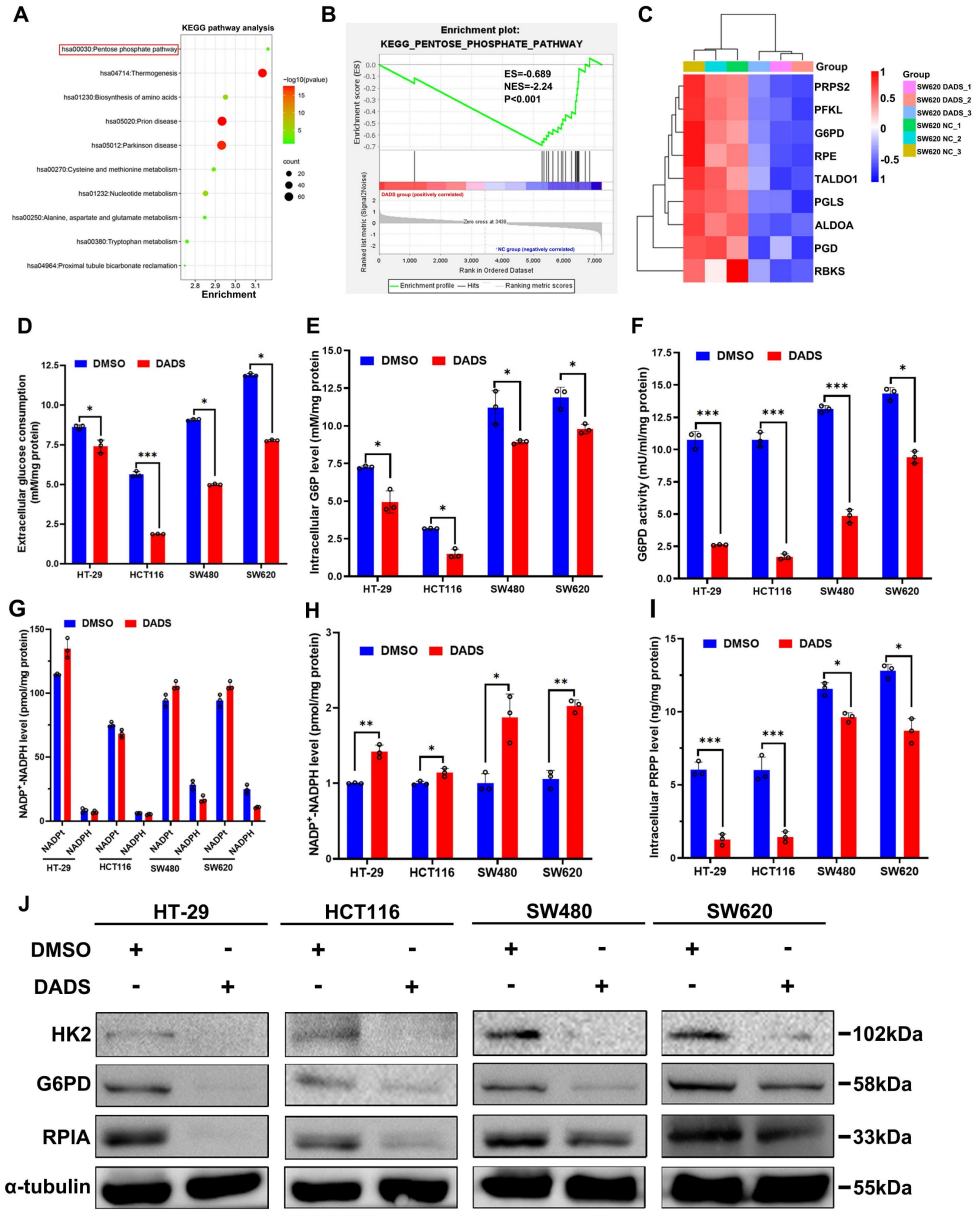
** DADS mitigates the PPP activity and PRPP production in CRC cells.** (**A**) KEGG pathway enrichment analysis of 141 DEPs between DADS-treated and control SW620 cells. (**B**) GSEA analysis of PPP-related genes enriched in control group. (**C**) Heatmap analysis of the PPP-related DEPs between DADS-treated and control SW620 cells. (**D-F**) The levels of glucose consumption, intracellular G6P levels and G6PD activities in the indicated cells. (**G-H**) The levels of intracellular NADP^+^ and NADPH and the ratios of NADP^+^/NADPH in the indicated cells. (**I**) The levels of PRPP in the indicated cells. (**J**) Western blot analysis of HK2, G6PD and RPIA expression in the indicated cells. Data are representative images or expressed as the mean ± SD of each group of samples from three separate experiments. *P<0.05, **P<0.01, ***P<0.001.

It is well known that the DNA damage repair may restore the DNA stability and function and is dependent on an efficient dNTP pool 23. Coincidentally, that Gene Set Enrichment Analysis (GSEA) analysis of the DEPs from the DADS-treated CRC cells unveiled that the DEPs were involved in purine- and pyrimidine-metabolism were much enriched (**Figure S3A**) and the GO analysis indicated that the changes in protein expression induced by DADS were closely associated with nucleotide synthesis (**Figure S3B**). Actually, DADS treatment significantly down-regulated the expression of nucleotide synthesis-related enzymes, such as ADSL (adenylosuccinate lyase), ATIC (formyltransferase/IMP cyclohydrolase), GMPS (Guanine Monophosphate Synthase) in CRC cells, compared to the vehicle DMSO group (**Figure S3C**), suggesting that DADS might suppress nucleotide synthesis and the DNA damage repair. Given that nucleotide synthesis heavily relies on the pentose phosphate pathway (PPP) to produce 5-phosphate ribose (R5P) 24, we therefore examined the effect of DADS treatment on the PPP activity in CRC cells. The GSEA and Kyoto Encyclopedia of Genes and Genomes (KEGG) pathway analysis revealed that DADS treatment significantly altered the expression of many proteins involved in the PPP in CRC cells (**Figure 3A-C**). Therefore, we hypothesized that DADS treatment might suppress the PPP activity to decrease nucleotide synthesis, thereby inhibiting DNA damage repair and inducing cell apoptosis.

To test this hypothesis, CRC cells were treated with vehicle or DADS, and the levels of extracellular glucose consumption, intracellular glucose-6-phosphate (G6P) levels, glucose-6-phosphatedehydrogenase (G6PD) activity, and NADPH levels in CRC cells were examined. We observed that DADS treatment significantly mitigated the levels of glucose consumption, intracellular G6P levels, intracellular G6PD activities, but increased the NADP^+^/NADPH ratios in CRC cells (P<0.05, **Figure 3D-H**). Consequently, DADS treatment significantly decreased the levels of intracellular PRPP (5-phosphate ribose-1-pyrophosphate) in CRC cells (P<0.05, **Figure 3I**). Similarly, DADS treatment obviously down-regulated the relative levels of hexokinase 2 (HK2), G6PD, and ribose 5-phosphate isomerase A (RPIA) expression in CRC cells (**Figure 3J**). IHC analysis revealed lower levels of HK2, G6PD and RPIA expression in the DADS-treated xenograft tumors relative to the control tumors (**Figure S3D**). Additionally, treatment with G6PD AG1 activator (a selective G6PD activator) significantly increased G6PD activities and reduced γ-H_2_AX and 53BP1 expression, while DADS treatment reversely reduced G6PD activities and partially rescued γ-H_2_AX and 53BP1 expression in CRC cells (P<0.05, **Figure S3E-F**). Together, these data suggested that DADS treatment suppressed the activation of PPP and increased DNA damage, resulting in apoptosis of CRC cells.

### DADS reduces the PPP activity by targeting POU2F1 in CRC cells

**Figure 4:**
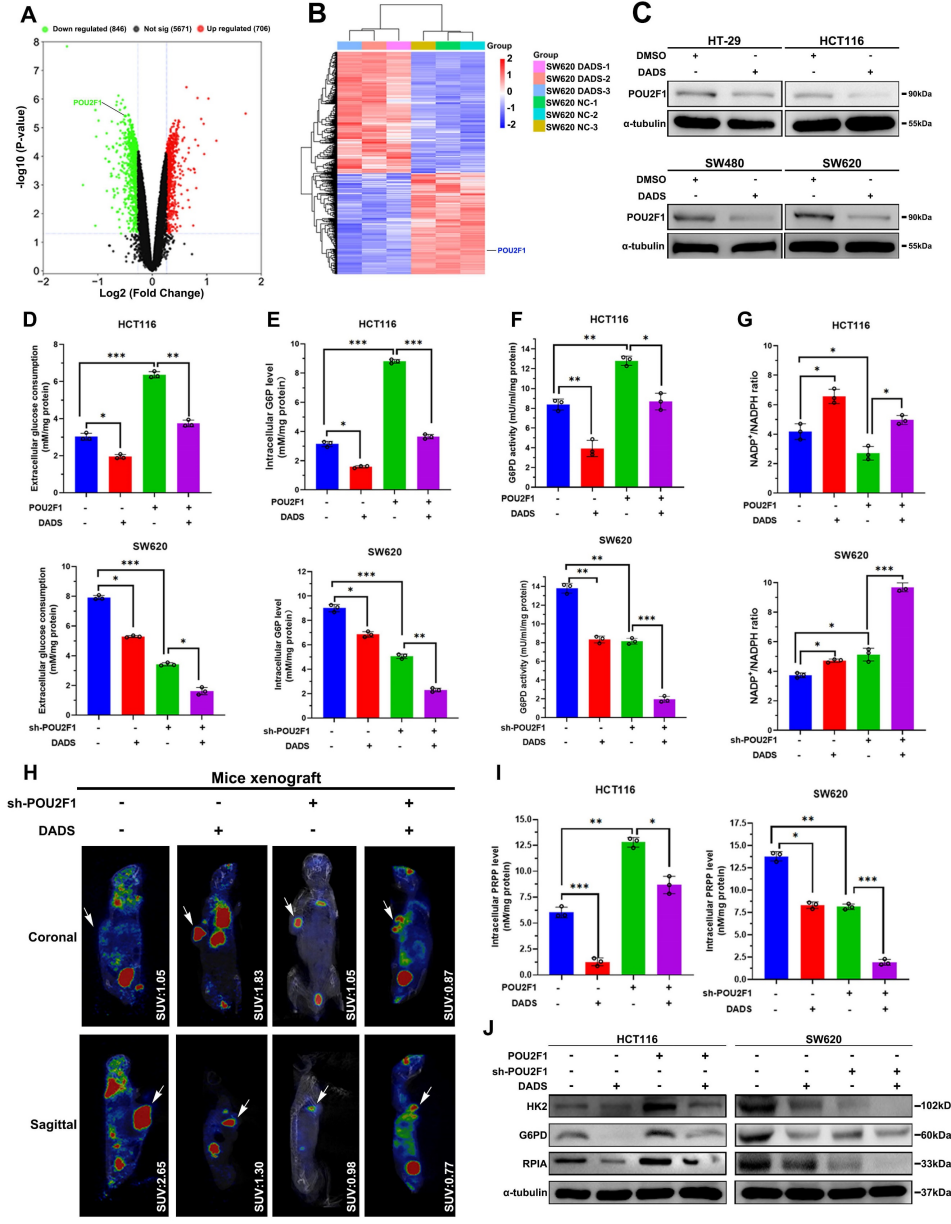
** DADS inhibits the PPP activity, dependent on reducing POU2F1 protein in CRC cells.** (**A-B**) Volcano plot and heatmap analyses displayed the location of POU2F1 in the down-regulated DEPs from the DADS-treated SW620 cells. (**C**) Western blot analysis revealed that DADS treatment reduced POU2F1 protein in the indicated cells. (**D-G**) The levels of glucose consumption, intracellular G6P levels, G6PD activities, and the ratios of NADP^+^/NADPH in indicated cells. (**H**) Representative images of ^18^F-FDG PET/CT scanning of wild-type or POU2F1 silencing SW620 tumors in the mice that had been treated vehicle or DADS (100 mg/kg) (n=5 per group). The SUV values in the region of interest (ROI) of individual mice were calculated. (**I**) The levels of PRPP in indicated cells. (**J**) Western blot analyses of the relative levels of HK2, G6PD and RPIA expression in the indicated cells. Data are representative images or expressed as the mean ± SD of each group of samples from three separate experiments. *P<0.05, **P<0.01, ***P<0.001.

Next, we explored the mechanisms underlying the actions of DADS treatment in suppressing the PPP activity in CRC cells. It was notable that POU2F1 was one of the down-regulated DEPs in the DADS-treated SW620 cells (**Figure 4A-B**). Interestingly, our previous research has shown that POU2F1 can promote the proliferation and oxaliplatin resistance of CRC cells by enhancing glycolysis and the PPP activity 7. Given the importance of POU2F1 in glycolysis and the PPP activity, we assessed whether DADS treatment could inhibit POU2F1 expression and the PPP activity in CRC cells. Actually, DADS treatment decreased POU2F1 protein levels in CRC cells (**Figure 4C**). Furthermore, DADS treatment not only significantly decreased glucose consumption, intracellular G6P levels and G6PD activities, but also significantly mitigated or abrogated the POU2F1 over-expression-increased glucose consumption, intracellular G6P levels and G6PD activities in CRC HCT116 cells.

In contrast, DADS treatment enhanced the POU2F1 silencing-decreased glucose consumption, intracellular G6P levels and G6PD activities in CRC SW620 cells (P<0.05 for all, **Figure 4D-F**). Moreover, DADS treatment abrogated the POU2F1 over-expression-decreased NADP^+^/NADPH ratios in HCT116 CRC cells while the same treatment synergistically further elevated the POU2F1 silencing-increased NADP^+^/NADPH ratios in SW620 CRC cells (P<0.05 for all, **Figure 4G**). The analysis of ^18^F-FDG PET/CT in tumor-bearing mice showed that that the maximum standard uptake values (SUV max) in the region of interest (ROI) were lower in mice with POU2F1 silencing tumors than in mice with wild-type tumors in the absence of DADS treatment and further reduced following DADS treatment (**Figure 4H**). While POU2F1 over-expression significantly elevated intracellular PRPP levels in HCT116 cells POU2F1 silencing dramatically decreased the PRPP levels in SW620 cells (P<0.05, **Figure 4I**). Importantly, DADS treatment significantly mitigated the positive enhancement of POU2F1 over-expression and deteriorated the inhibitory effect of POU2F1 silencing on the PRPP production in CRC cells. Similar patterns of HK2, G6PD, and RPIA expression were detected in the indicated CRC cells following DADS treatment by Western blot assays (**Figure 4J**). Additionally, DADS treatment significantly increased DNA damage and apoptosis (P<0.05, **Figure S4** and **Figure S5A-B**), and inhibited the proliferation of POU2F1 over-expressing CRC cells (P<0.05, **Figure S5C**). Consistently, DADS treatment significantly decreased the growth of POU2F1 over-expressing CRC in mice (P<0.05, **Figure S5D-F**). IHC analysis revealed lower levels of POU2F1, HK2, G6PD and RPIA expression in the DADS-treated xenograft tumors, relative to the control tumors (**Figure S6**). Collectively, these data indicated that DADS attenuated the PPP activity by reducing POU2F1 protein levels to promote DNA damage and apoptosis, ultimately causing growth inhibition *in vitro* and *in vivo*.

### DADS selectively promotes POU2F1 degradation through the K48-linked ubiquitination in CRC cells

**Figure 5:**
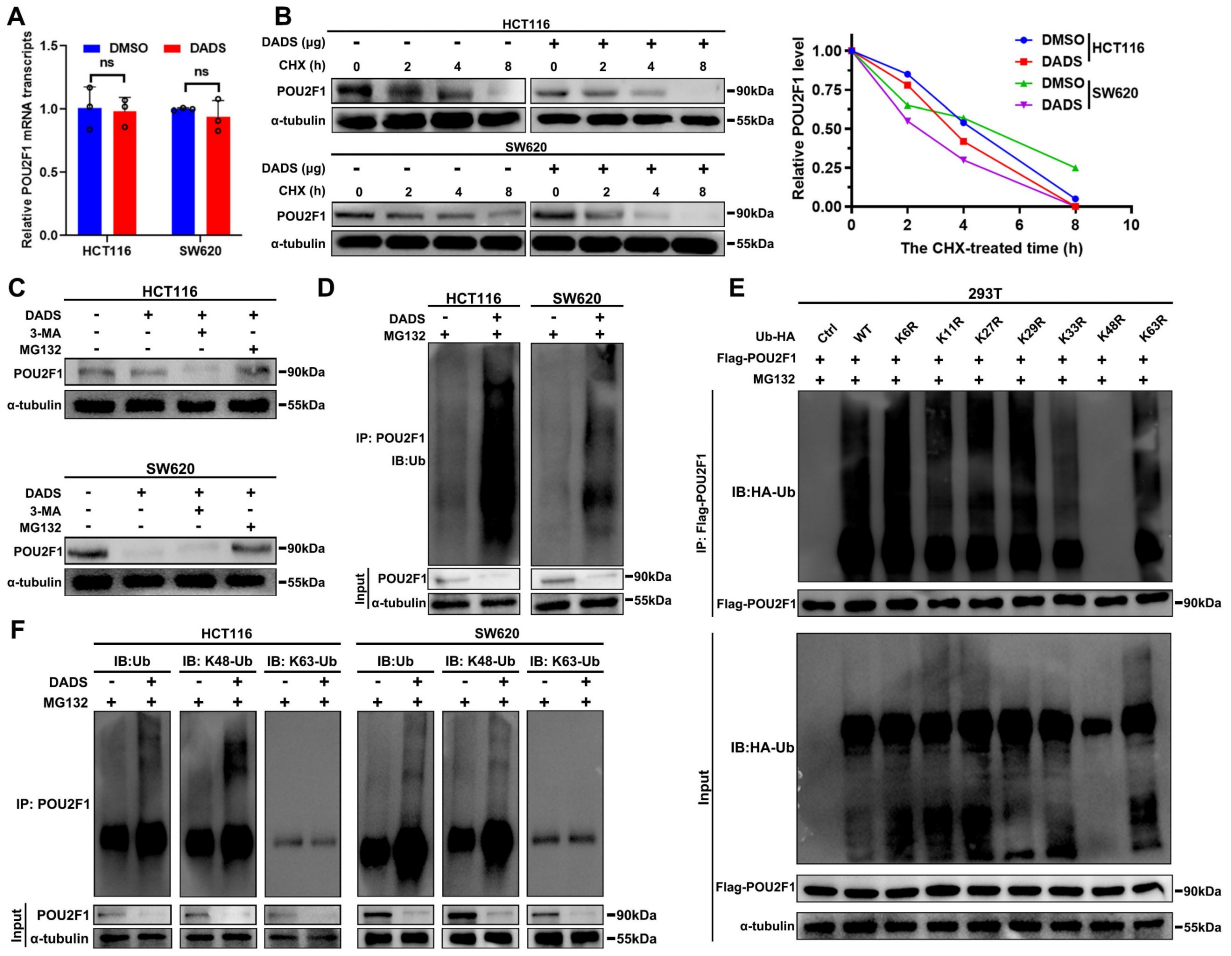
** DADS promotes the K48-linked POU2F1 ubiquitination and degradation.** (**A**) RT-qPCR analysis of the relative levels of POU2F1 mRNA transcripts in the indicated cells. (**B**) Western blot analysis of POU2F1 degradation in the indicated cells following treatment with vehicle or DADS in the presence or absence of CHX for the indicated time periods. (**C**) Western blot analysis of POU2F1 protein levels in the indicated cells following the indicated treatments. CRC(**D**) Co-IP detected the effects of DADS on POU2F1 protein ubiquitination in the indicated CRC cells in the presence of MG132CRC. (**E**) An* in vitro* ubiquitylation assay was performed using lysine-to-arginine Ub mutants (5 μg/ panel). The products were analyzed by Western blot using anti-Ub antibody. (**F**) Co-IP detected the effects of DADS on the K48- or K63-linked POU2F1 ubiquitination CRCin the indicated CRC cells. Data are representative images or expressed as the mean ± SD of each group of samples from three separate experiments. Ns: no significance.

Given that DADS treatment reduced the amount of POU2F1 protein in CRC cells (**Figure 4C**), we further investigated how DADS treatment regulated the POU2F1 expression. Firstly, DADS treatment did not significantly alter the relative levels of POU2F1 mRNA transcripts in CRC cells (P>0.05, **Figure 5A**). Notably, the DEPs induced by DADS treatment were involved in the process of protein stabilization and ubiquitin protein ligase binding (**Figure 1D**). We hypothesized that DADS may regulate the stabilization of POU2F1 protein in CRC cells. Actually, DADS treatment accelerated the decrease in the relative levels of PO2F1 protein in CRC cells in the presence of protein synthesis inhibitor CHX (cycloheximide) (**Figure 5B**), suggesting that DADS treatment might promote the degradation of POU2F1 protein in CRC cells.

To further elucidate how DADS treatment reduced the POU2F1 protein stability, CRC cells were treated with vehicle or DADS alone or together with MG132 (a proteasome inhibitor) or 3-MA (an inhibitor of lysosome-autophagy pathway) and their POU2F1 protein levels were analyzed by Western blot assays. The results exhibited that DADS treatment reduced the POU2F1 protein levels in both untreated CRC and 3-MA-treated CRC cells, but not in the MG132-treated CRC cells (**Figure 5C**). These data suggest that DADS treatment may promote the ubiquitin-proteasome degradation of POU2F1 protein in CRC cells. Indeed, DADS treatment enhanced the levels of POU2F1 ubiquitination in CRC cells (**Figure 5D**). Ub can be further ubiquitinated at 1 of the 7 lysine sites (K6, K11, K27, K29, K33, K48, and K63) to polyubiquitinate substrates by specific E3 Ub ligases 25. To explore the type of Ub chain generated on POU2F1, we mutated each of the lysine residues on Ub and evaluated their effect on POU2F1 ubiquitination and found that only the K48R (Lys48-Arg) mutation in Ub completely prevented POU2F1 polyubiquitination in 293T cells (**Figure 5E**). Moreover, DADS -mediated POU2F1 ubiquitination was linked to K48, but not to K63 Ub in HCT116 and SW620 cells (**Figure 5F**). Thus, DADS treatment was like to promote POU2F1 K48-linked ubiquitination and degradation through the proteasome pathway.

### DADS up-regulates TRIM21 expression, through the K272 in POU2F1 to induce the POU2F1 K48-linked ubiquitination and proteasomal degradation in CRC

**Figure 6:**
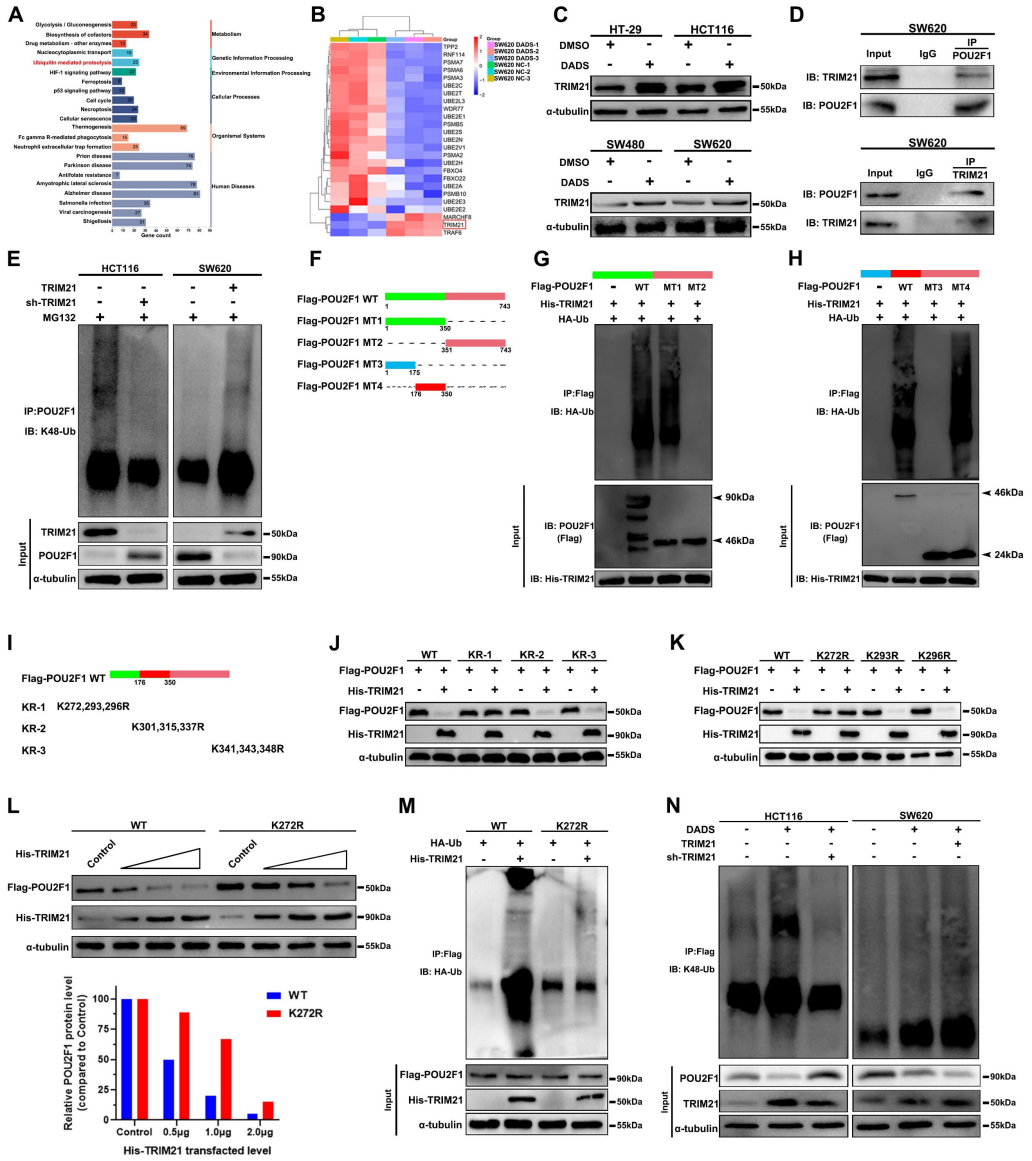
** DADS enhances TRIM21 expression and the K48-linked POU2F1 K272 ubiquitination and degradation in CRC cells.** (**A-B**) The KEGG pathway annotation and heatmap analysis displayed the distribution of up-regulated TRIM21 expression in the DEPs of DADS-treated SW620 cells. (**C**) Western blot analyses of the relative levels of TRIM21 expression in the indicated cells. (**D**) Co-IP revealed the direct interaction between TRIM21 and POU2F1 proteins in CRC cells. (**E**) Co-IP detected the effects of TRIM21 on POU2F1 protein ubiquitination in the indicated cells. (**F-H**) CO-IP analyses unveiled that the 176-350 region of POU2F1 protein was crucial for the TRIM21-mediated POU2F1 ubiquitination in CRC cells. (**I-K**) Western blot analyses of the mutants indicated that the K272 was an acceptor of POU2F1 for the TRIM21-mediated POU2F1 ubiquitination. (**L-M**) The K272R mutant of POU2F1 abolished the TRIM21-mediated POU2F1 degradation. (**N**) Co-IP detected the effects of TRIM21 on DADS-mediated POU2F1 ubiquitination in the indicated cells.

Next, we examined molecular mechanisms by which DADS treatment enhanced the POU2F1 ubiquitination in CRC cells. The KEGG pathway enrichment analysis unveiled that the DEPs induced by DADS treatment were enriched in ubiquitin-mediated proteolysis in CRC cells (**Figure 6A**). The E3 ligase TRIM21 expression was upregulated in the DADS-treated CRC cells (**Figure 6B-C**). As well known, TRIM proteins, as a kind of RING-type E3 ubiquitin ligases, can transfer ubiquitin to target proteins for degradation 26. Accordingly, we reasoned that TRIM21 might be responsible for the K48-linked ubiquitination of POU2F1. Evidently, the levels of POU2F1 expression were negatively correlated with TRIM2F1 expression in 458 colon adenocarcinoma cases in The Cancer Genome Atlas (TCGA) database (r=-0.1568, P<0.001, **Figure S7A**). Secondly, Co-IP assays exhibited that TRIM21 protein interacted with POU2F1 in CRC cells (**Figure 6D** and **Figure S7B**). Furthermore, while TRIM21 over-expression enhanced POU2F1 K48-linked ubiquitination to reduce the levels of POU2F1 protein, TRIM21 silencing had an opposite effect in CRC cells (**Figure 6E**). More importantly, re-introduction of TRIM21 expression significantly rescued the POU2F1 ubiquitination in the TRIM21-silenced HCT116 cells, while TRIM21 silencing obviously attenuated the POU2F1 ubiquitination in the TRIM21 over-expressing SW620 cells (**Figure S7C**).

TRIM21 promotes its targets for degradation by the ubiquitination of lysine residues in the substrates 27. Therefore, we determined the importance of lysines in the TRIM21-mediated POU2F1 protein ubiquitination. To identify which lysine(s) in POU2F1 is required for TRIM21-mediated POU2F1 ubiquitination and subsequent degradation, we firstly constructed different structural domains of POU2F1 mutants (**Figure 6F**), and the results revealed that the region (176-351aa) of POU2F1 was essential for the TRIM21-mediated POU2F1 ubiquitination in CRC cells (**Figure 6G-H**). Subsequently, we divided these lysines in the region (176-351aa) into three subregions and mutated each of them to arginine (R) (**Figure 6I**). We found that the mutations of all lysines in the KR-1 group, but not those in other groups, completely abolished the TRIM21-mediated POU2F1 degradation, suggesting that lysines (K272, 293 and 296) in this region of POU2F1 may be critical for the TRIM21-mediated POU2F1 ubiquitination in CRC cells (**Figure 6J**). Further point mutations revealed that the mutation of K272R, but not the other lysine residues, almost completely abolished the TRIM21-mediated POU2F1 degradation in CRC cells (**Figure 6K-L**). Similarly, the mutation of K272R in POU2F1 also abrogated the TRIM21-mediated POU2F1 ubiquitination in CRC cells (**Figure 6M**).

Given that TRIM21 promoted POU2F1 ubiquitination and degradation, we next investigated whether TRIM21 contributed to the DADS-induced POU2F1 ubiquitination. As shown in **Figure 6N**, DADS treatment obviously up-regulated TRIM21 protein expression and enhanced POU2F1 ubiquitination, decreasing POU2F1 protein levels in CRC HCT116 cells, which were reduced in the TRM21-silencing HCT116 cells. Furthermore, DADS treatment enhanced the TRIM21-increased POU2F1 ubiquitination, reducing POU2F1 protein levels in the TRIM21 over-expressing SW620 cells. These indicated that DADS treatment promoted POU2F1 ubiquitination and degradation by increasing TRIM21 protein expression in CRC cells. Together, these data indicated that the K272 in POU2F1 was the key acceptor site for the TRIM21-mediated POU2F1 K48-linked ubiquitination and subsequent degradation in CRC cells.

### DADS inhibits the PI3K/AKT signaling to up-regulate TRIM21 expression and trigger POU2F1 ubiquitination and degradation in CRC cells

**Figure 7:**
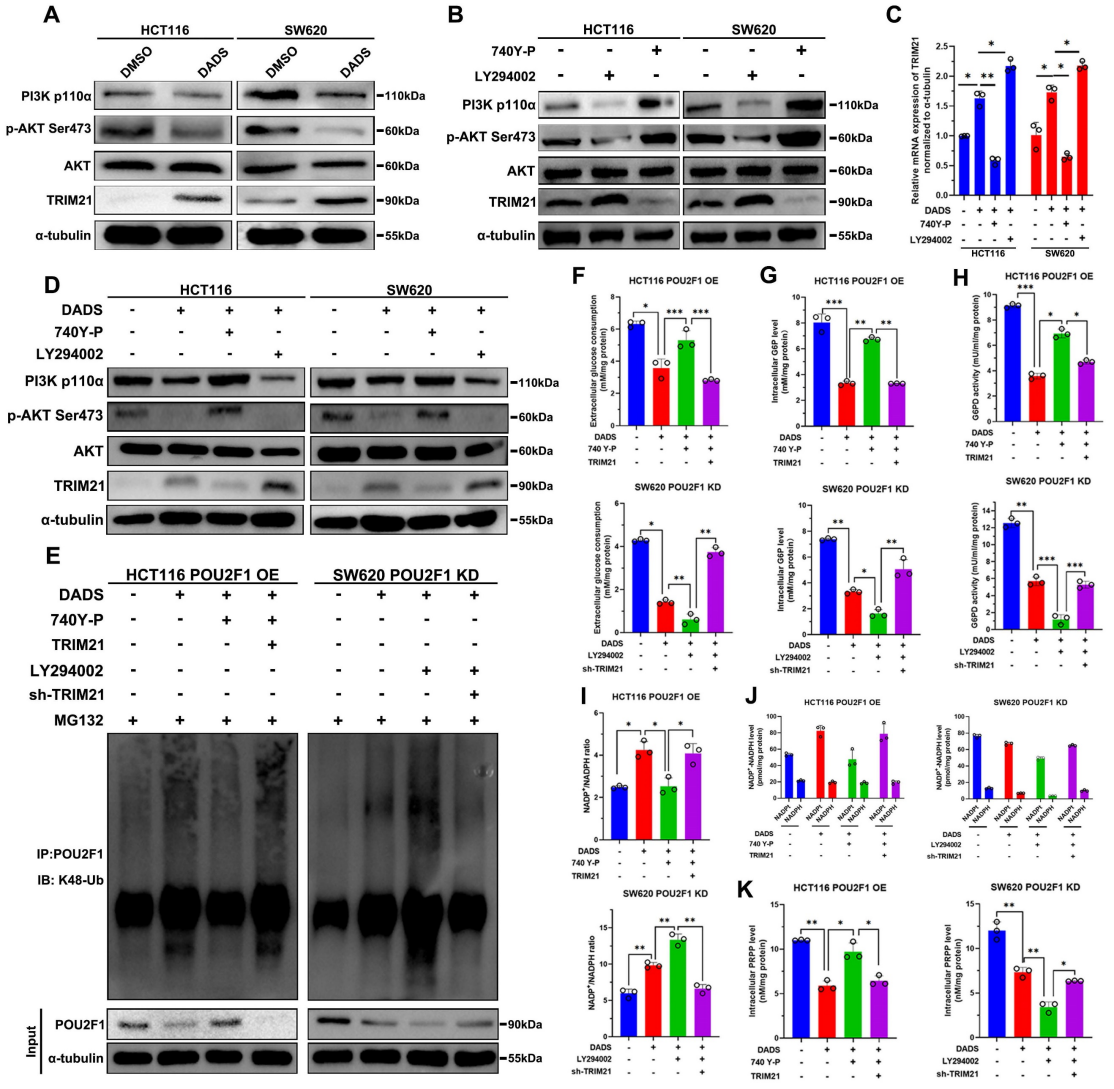
** DADS up-regulates TRIM21 expression to reduce the PPP activity by attenuating the PI3K/AKT signaling in CRC.** (**A**) Western blot analyses indicated that DADS up-regulated TRIM21 expression and attenuated the PI3K/AKT activation in the indicated cells. (**B**) Western blot detected the effects of the PI3K/AKT signaling activator (10 μM 740 Y-P) or inhibitor (30 μM LY294002) on TRIM21 expression in the indicated cells. (**C-D**) RT-qPCR and Western blot analyses detected TRIM21 expression in the indicated CRC cells following treatment with vehicle or DADS alone or combination with CRC740 Y-P or LY294002. (**E**) Co-IP detected the effects of DADS, 740 Y-P, LY294002 and TRIM21 on POU2F1 protein ubiquitination in POU2F1 over-expressing or silencing CRC cells. (**F-K**) The levels of glucose consumption, intracellular G6P levels, G6PD activities, the ratios of NADP^+^/NADPH, intracellular NADPH levels, and PRPP levels in the indicated cells. Data are representative images or expressed as the mean ± SD of each group of samples from three separate experiments. *P<0.05, **P<0.01, ***P<0.001.

How did DADS enhance the TRIM21-mediated POU2F1 ubiquitination? A previous study has reported that TRIM21 was negatively regulated by the PI3K/AKT signaling 28. Our previous study has shown that DADS treatment attenuates the PI3K/AKT signaling in CRC cells 14. Accordingly, we tested whether DADS through inhibiting the PI3K/AKT pathway up-regulated TRIM21 expression in CRC cells. The results displayed that comparison with the DMSO-treated control cells, DADS treatment up-regulated TRIM21 protein expression, but not AKT expression, and reduced PI3Kp110α and AKT Ser473 phosphorylation in CRC cells (**Figure 7A**). Furthermore, treatment with 740Y-P to activate the PI3K/AKT signaling decreased TRIM21 expression while treatment with LY294002 to inhibit the PI3K/AKT signaling increased TRIM21 expression in CRC cells (P<0.05, **Figure 7B**). Consistently, DADS treatment synergistically increased the LY294002-enhanced TRIM21 expression, but decreased TRIM21 expression in the 740Y-P-treated CRC cells (**Figure 7C-D**). Therefore, DADS up-regulated the expression of TRIM21 by attenuating the PI3K/AKT signaling in CRC cells.

Finally, we tested how DADS treatment modulated POU2F1 ubiquitination in POU2F1 over-expressing HCT116 cells and POU2F1 silencing SW620 cells. Comparison with the MG132-treated control cells, DADS treatment enhanced POU2F1 ubiquitination, which were attenuated by 740Y-P treatment, but enhanced by induction of TRIM21 over-expression in POU2F1 over-expressing HCT116 cells (**Figure 7E**). Furthermore, DADS treatment also increased POU2F1 ubiquitination, which was dramatically enhanced by LY294002 treatment in POU2F1 silencing SW620 cells. In contrast, TRIM21 silencing attenuated the DADS and LY294002-enhanced POU2F1 ubiquitination in POU2F1 silencing SW620 cells (**Figure 7E**). As a result, 740Y-P treatment partially rescued the DADS-decreased glucose consumption, intracellular G6P levels and G6PD activities, which were significantly mitigated or abrogated by inducing TRIM21 over-expression in POU2F1 over-expressing HCT116 cells (P<0.05 for all, **Figure 7F-H**). In contrast, treatment with LY294002 to inhibit the PI3K/AKT signaling synergistically decreased the DADS-reduced glucose consumption, intracellular G6P levels and G6PD activities, while TRIM21 silencing reversely increased glucose consumption, intracellular G6P levels and G6PD activities in POU2F1 silencing SW620 cells. Additionally, 740Y-P treatment abrogated the DADS-increased NADP^+^/NADPH ratios, which was rescued by TRIM21 over-expression in POU2F1 over-expressing HCT116 cells (P<0.05 for all, **Figure 7I-J**). Conversely, LY294002 treatment enhanced the DADS-increased NADP^+^/NADPH ratios, which was abolished by inducing TRIM21 silencing in POU2F1 silencing SW620 cells. Moreover, 740Y-P treatment partially rescued the DADS-decreased intracellular PPAR levels, which was abrogated by inducing TRIM21 over-expression in POU2F1 over-expressing HCT116 cells. Conversely, LY294002 treatment further reduced the DADS-decreased intracellular PPAR levels, which was partially rescued by inducing TRIM21 silencing in POU2F1 silencing SW620 cells **(**P<0.05 for all, **Figure 7K**). These data indicated that DADS up-regulated TRIM21 expression by inhibiting the PI3K/AKT signaling to enhance POU2F1 ubiquitination, but to inhibit the PPP flux in CRC cells. Functionally, comparison with the control cells, DADS treatment significantly inhibited the proliferation of POU2F1 over-expressing HCT116 cells, which was dramatically rescued by 740Y-P treatment (P<0.05, **Figure S7D**). Induction of TRIM21 over-expression significantly mitigated the 740Y-P treatment-enhanced proliferation of POU2F1 over-expressing HCT116 cells following DADS treatment. While DADS treatment significantly decreased the proliferation of POU2F1 over-expressing SW620 cells LY294002 treatment significantly enhanced the inhibitory effect of DADS on the proliferation of both POU2F1 and/or TRIM21 silencing SW620 cells (P<0.05, **Figure S7D**). Collectively, our data indicated that DADS inhibited the growth of CRC cells by inhibiting the PI3K/AKT/TRIM21/POU2F1 axis.

## Discussion

Various oncogenic pathways contribute to the development and progression of colorectal cancer (CRC). Hence, identifying the key molecular driver events associated with cancer growth will uncover new therapeutic targets and allow for developing new therapeutic strategies for the management of CRC patients 2. Previous studies have demonstrated that DADS has potent anti-tumor activities in various types of malignant tumors, such as neuroblastoma, breast cancer, CRC, lung cancer, and gastric cancer 16, 29-32. A previous study has indicated that DADS treatment causes DNA damage and apoptosis of cancer cells, contributing to its anti-cancer effects 33. In this study, we found that DADS treatment inhibited the growth of CRC cells* in vitro* and *in vivo* by inducing their DNA damage-related apoptosis. Hence, DADS may provide a novel DNA damage-based cancer therapy for CRC.

The toxin-conjugated antibodies can release toxin to induce DNA damage and microtubule inhibition, leading to the targeted tumor cell apoptosis and can kill surrounding cancer cells through the bystander effect 34. A previous study has reported that high levels of intracellular ROS can activate intrinsic apoptosis pathway by increasing mitochondrial permeability and inhibiting the PPP flux in cancer cells 35. In this study, we found that DADS treatment failed to increase the intracellular ROS levels and reversed the inhibition of GSK2795039 (an inhibitor of NADPH oxidase 2) on DNA damage, indicating that DADS treatment enhanced DNA damage in CRC cells, independent of elevating intracellular ROS levels. However, our proteomic results showed that the DEPs in DADS-treated group were mainly associated with the DSB (DNA double strand break) repair, DNA replicate, and DNA biosynthetic pathways. As well known, the process of DNA replication is constantly challenged by various intrinsic and extrinsic stresses throughout the genome 36. The most frequent events of DNA damage are DSB, which severely influenced the cell survival and proliferation 37. Actually, the DSB repair, and DNA replication closely depended on DNA biosynthesis, on the one hand, but the process of DNA replication includes duplex unwinding, followed immediately by DNA synthesis, on the other hand, and DNA synthesis is disturbed in damaged DNA regions, in replication slow zones, or as a result of insufficient nucleotide level 38. Several lines of evidence have demonstrated that enhanced nucleotide metabolism can promote DNA damage repair to confer growth inhibition. For instance, activation of glycolysis, especially for the PPP flux, can enhance the nucleotide metabolism and promote DNA damage repair 39-41. Interestingly, our proteomic analysis revealed that the DEPs involved in the PPP activation were highly down-regulated in the DADS-treated CRC cells, and DADS treatment decreased the NADPH and PRPP production and nucleoside metabolism, thus suppressing DNA damage repair, and inducing and apoptosis and growth inhibition of CRC cells.

In general, the PPP flux is particularly active in tumor cells to provide energy and nutrients to support their growth 42. Interestingly, our previous findings have shown that POU2F1 silencing specifically inhibits glycolysis and the PPP activity to suppress the ROS-mediated DNA damage and apoptosis, promoting the proliferation and oxaliplatin resistance in CRC cells 7, suggesting that POU2F1 may be a promising therapeutic target. In this study, we observed that DADS treatment inhibited the PPP flux to induce DNA damage and cell apoptosis by targeting POU2F1 protein stability. However, it remains unclear how DADS affects the POU2F1 protein stability. Ubiquitin-mediated proteasomal degradation is crucial for regulating protein stability and functions 43. we found that DADS treatment accelerated POU2F1 degradation by increasing its K48-linked ubiquitination in CRC cells. Consistently, DADS treatment suppressed the PRPP synthesis and nucleoside metabolism, inhibiting the DNA damage repair, and the growth of CRC cells.

Mechanistically, we found that DADS treatment up-regulated TRIM21 expression in CRC cells, accompanied by enhancing POU2F1 ubiquitination and degradation. TRIM21 is a RING finger domain-containing E3 ubiquitin ligase and belongs to the tripartite motif (TRIM) family. Previous studies have reported that TRIM proteins can positively or negatively regulate the process of carcinogenesis, including the DNA damage response pathway 44, 45. However, the precise role of TRIM21 in the pathogenesis of CRC remains under debate. It is notable that TRIM21 expression is up-regulated in a broad spectrum of cancers, including glioma, breast cancer and nasopharyngeal carcinoma (NPC) 27, 46-48. Surprisingly, we have found that TRIM21 is essential for PHB ubiquitination degradation in nasopharyngeal carcinoma (NPC) 49. Conversely, TRIM21 expression is down-regulated in CRC and negatively regulates intestinal epithelial carcinogenesis 50, and down-regulated TRIM21 expression is associated with enhancement of carcinogenesis and worse prognosis of hepatocellular carcinoma and breast cancer 51, 52. Thus, the levels of TRIM21 expression may vary in different types of malignant tumors. More importantly, we found that TRIM21 interacted with POU2F1 protein and promoted the K48-linked ubiquitination of POU2F1 at K272 site and degradation in CRC cells. Our findings suggest that TRIM21 may be a tumor suppressor and new therapeutic target for intervention of CRC.

A previous study has reported that the TRIM21 expression levels are negatively correlated with PI3K activity in various human cancers, implying that TRIM21 may act as a potential tumor suppressor 28. Coincidentally, we have shown that DADS inhibits the Rac1-mediated epithelial-mesenchymal transition by blocking the PI3K/AKT pathway 14. In this study, we found that DADS treatment inhibited the activation of PI3K/AKT signaling to enhance TRIM21 protein expression and the K48-linked POU2F1 K272 ubiquitination and subsequent degradation in CRC cells. These findings were consistent with the notion that activation of the PI3K/AKT signaling down-regulates TRIM21 expression 28. Although AKT downstream substrates (FOXO3A) may contribute to the PI3K/AKT/mTORC2-regulated TRIM21 expression, we did not find an FOXO3A binding sequence in the TRIM21 promoter 28. However, a previous study has pointed out that AKT binds to and phosphorylates PFKP (PFK1 platelet isoform) at S386 to inhibit the binding of TRIM21 E3 ubiquitin ligase to PFKP and the subsequent TRIM21-mediated PFKP polyubiquitylation and degradation 53. More interestingly, a recent study has discovered that TRIM21 knockout decreases the expression of PI3K phosphorylation and AKT expression 54. Apparently, there is a feedback loop between the PI3K/AKT pathway and TRIM21 expression. However, the precise regulation between the PI3K/AKT signaling and TRIM21 expression remains to be further investigated.

In conclusion, we herein report that DADS treatment inhibits the PPP flux by targeting POU2F1 protein stability in a TRIM21-dependent manner, and consequently decreasing PRPP generation and nucleotide synthesis. This in turn enhances DNA damage and growth inhibition in CRC cells. Collectively, our findings may provide new insights into the interaction among DADS, cell metabolism, and DNA damage, and also suggest that DADS can be a safe natural agent for CRC treatment.

## Materials and Methods

### Specific reagents

The specific reagents included the primary antibodies against AKT, POU2F1, and TRIM21 (Abcam, London, UK); against PI3K^p100α^, γ-H_2_AX, 53BP1 and p-AKT^Ser473^ (Cell Signaling Technology, Danvers, MA, USA); against α-tubulin, BCL-2 and BAX (Proteintech, Chicago, USA); horseradish peroxidase (HRP)-conjugated goat anti-rabbit IgG or goat anti-mouse IgG (Beyotime, Shanghai, China). GSK2795039, 3-MA and DADS (oil, ≥98%, and 1.008 g/mL) were purchased from Sigma, (Saint Louis, Missouri, USA), and the DADS was fully dissolved in Tween 80 and diluted at 1:100 in physiological saline and stored in a -20°C freezer. Additionally, 740Y-P, LY49002, MG132, AG activator 1 and cycloheximide as well as AV-153, an antimutagenic molecule were obtained from Selleckchem, Houston, USA and MedChenExpress, Monmouth Junctio, USA, respectively.

### Label-free quantitative proteomic analysis

SW620 cells were treated with vehicle or DADS for two days and collected for protein extraction. The protein samples were digested with trypsin and subjected to label-free quantitative proteomic analysis including HPLC fractionation, LC-MS/MS and data analysis by Shanghai OE Biotech (China). The study identified differentially expressed proteins (DEPs) using a fold change of ≥1.2 or ≤ -1.2 and a p-value <0.05, determined by R software. The proteomic profiles of SW620 cells treated with DADS were analyzed using R software and presented in a heatmap. The distribution of top DEPs was visualized using Volcano maps created with the ggplot2 package. The cases of CRC in the Molecular Signature Database were divided into two groups: scramble and sh-POU2F1, based on the median value of POU2F1 expression. The GSEA software (http://www.broadinstitute.org/gsea/index.jsp) was used to analyze the transcriptome profiles in gene sets of c2.kegg.v6.0.symbols.gmt between the two groups.

### Plasmid construction and transfection

The plasmid for the expression of POU2F1 was made in our laboratory previously 7 and added with a Flag-tag. The plasmids for the expression of His-TRIM21, HA-Ub WT and mutant K6, 11 27, 29, 33, 48 and 63 were purchased from GENE (Hong Kong, China). All constructs were confirmed by DNA sequencing. HEK293 cells were transfected with individual types of plasmids using Lipofectamine 3000 reagent (Invitrogen, Waltham, MA, USA), according to the manufacturer's instructions.

Similarly, CRC cells were transfected with control scramble shRNA or POU2F1-specific shRNA using Lipofectamine 3000, as our previous description 7. In addition, CRC cells were also transfected with control shRNA: 5′-UUCUCCGAACGUGUCACGUTT-3′ and 5′-ACGUGACACGUUCGGAGAATT-3′; shTRIM21: 5′-GACUUCACCUGUUCUGUGATT-3′ and 5′-UCACAGAACAGGUGAAGUCTT-3′. Moreover, CRC cells were transfected with the control or plasmids for the expression of POU2F1 or TRIM21 using lipofectamine 3000. He transfected cells were treated with 2 µg/ml of puromycin (Sigma, Saint Louis, Missouri, USA) to generate stable POU2F1 or TRIM21 over-expressing or POU2F1 or TRIM21-silencing cells.

### Cell viability and colony formation assays

The proliferation of each group of CRC cells was examined by CCK-8 assays. Briefly, each group of cells (5000 cells/well) were cultured in 96-well plates and treated in triplicate with DADS at 45 μg/ml (SW620) or 35 μg/ml (HCT116) for 0, 24, 48 or 72 h 55. After reaction with CCK-8 reagents during the last 3-h culture, the cell viability in each well was measured for the absorbance at 450 nm in a microplate reader. To determine the clonogenicity of individual groups of cells, the cells (2000 cells/dish) were cultured in triplicate in 6-cm dishes for 10-14 days, fixed with methanol and stained with 0.1% crystal violet. The visible colonies were counted in a blinded manner.

### *In vivo* and* in vitro* ubiquitination assays

Both *in vivo* and *in vitro* ubiquitination assays were performed as previous report 56. Briefly, 293T cells were transfected with the plasmids for the expression of Flag-POU2F1, His-TRIM21 and HA-ubiquitin for two days. Their cell lysates were prepared and boiled in 1% SDS RIPA buffer for 5 min. The same number of proteins from each group was immunoprecipitated with anti-HA microbeads and after being washed, the bound proteins were resolved by Western blot using anti-HA and anti-Flag to view POU2F1 ubiquitination. To detect the *in vitro* POU2F1 ubiquitination, Flag-POU2F1, His- UbcH5a, and HA-ubiquitin that were purified from *E. coli* were reacted with purified E1 (Boston Biochem, USA), E2, ubiquitin, His-flag- PIPKIγi2, GST-Smurf1, and 2 mM ATP in a final volume of 30 μl at 30 °C for 2 h. The reactions were stopped by adding 180 μl of denaturing lysis buffer (50 mM Tris-HCl, 150 mM NaCl (pH7.5), 0.1% Triton X-100, 1 mM EDTA containing 1% SDS and 1% deoxycholate) and heated at 70 °C for 20 min. After diluted, the POU2F1 proteins were immunoprecipitated using anti-Flag and protein A/G beads for 2 h at 4 °C. After being washed, the bound ubiquitinated POU2F1 proteins were analyzed by IB using an anti-Ubiquitin antibody.

### Measurements of glucose consumption, lactate production, intracellular G6P levels, G6PD activities and NADPH levels

Following transfection, the CRC cells (1×10^6^ cells/well) were incubated for 24 h. Glucose levels in the supernatants of cultured cells and cell lysates were measured using the Glucose Colorimetric Assay Kit (BioVision, Milpitas, USA) and the levels of lactate in the supernatants of cultured cells were quantified by an automatic analyzer. The levels of intracellular G6P in cell lysates were analyzed using a G6P Assay kit (BioVision, Milpitas, USA). In addition, the levels of intracellular G6PD enzymatic activity and NADPH in cell lysates were determined using G6PD Assay Kit (ab102529, Abcam, UK) and Amplite TM Colorimetric NADP/NADPH Ratio Assay Kit (ab65349, Abcam, UK), respectively.

### Flow cytometry

The SW620 and HCT116 cells were treated with vehicle or DADS at 45 μg/ml (SW620) or 35 μg/mL (HCT116) for 48 h. Their intracellular ROS levels were quantified using DCFH_2_ in a Cytomics FC500 Flow Cytometry System (Beckman Coulter, Krefeld, Germany).

The frequency of apoptotic cells was analyzed by flow cytometry 57. Briefly, following treatment, the cells in each group were stained with Annexin V-FITC and propidium iodide (PI) and the percentages of apoptotic cells were analyzed by flow cytometry. All flow cytometric data were analyzed using Flow Jo software.

### Measurement of PRPP levels

Following treatment with DADS for two days, intracellular PRPP levels were analyzed using a 5-phosphate ribose 1 pyrophosphate (PRPP) ELISA kit (Chemical Book, CB75526517), following the manufacturer's instructions.

### TUNEL assay

The frequency of apoptotic CRC cells was determined by terminal dUTP transferase nick end labeling (TUNEL) assay using a specific kit (ab66108, Abcam), per the manufacturer's protocol. Briefly, after the DADS treatment, the CRC cells were fixed with 4% paraformaldehyde for 15 min at room temperature, washed with PBS, and permeabilized with 0.1% Triton X-100 for 5 min at room temperature. Subsequently, the CRC cells were stained with 50 μl TUNEL reaction mixture at 37°C for 60 min and washed with PBS and photoimaged under a confocal microscopy (LSM510 META, ZEISS, Germany).

### *In vivo* xenograft model of CRC and PET/CT study

Female BALB/c nude mice aged 4-6 weeks were obtained from the Animal Experiment Center of Hunan Cancer Hospital and housed in a specific pathogen-free room in the animal research center of our hospital. Subsequently, individual mice were subcutaneously injected with POU2F1-silencing or scrambler siRNA-transfected SW620 cells (5×106 cells/mice, n= 7/group). After the tumor volume reached 200 mm3, the mice were injected i.p. with normal saline or DADS (100 mg/kg) every other day for 4 weeks 55. Tumor volumes were measured every three days from the start of treatment until the mice were euthanized in a blinded manner. The formula V=1/2L×W2 was used to calculate tumor volumes (V), where W represents the largest tumor diameter and L represents the second largest tumor diameter. The day before euthanasia, mice were individually starved for six hours and underwent microPET/CT imaging for one hour using the ^18^F-FDG probe at a dose of 6 µCi/g body weight. PET-CT (positron emission tomography-computed tomography) was used to analyze the data and determine the standardized uptake value (SUV) in the region of interest (ROI). After euthanizing the mice, their tumors were dissected, photoimaged, and weighed. Histology and IHC were performed on the tumor tissues.

### Additional materials and method

The supplementary materials provide additional details on the other materials and methods used. The primer and antibody information are listed in the **Table S1, Table S2**.

### Statistical analysis

The data are presented as the mean ± standard deviation (SD). The difference between groups was analyzed using Student's t-test. All statistical analyses were performed using SPSS version 22.0 (SPSS, Chicago, IL, USA). A p-value of less than 0.05 was considered statistically significant.

### Availability of data and materials

The public datasets analyzed during the current study are available in the repositories listed below:

*•* The Cancer Genome Atlas

TCGA-COAD: https://portal.gdc.cancer.gov/projects/TCGA-COAD

## Supplementary Material

Supplementary figures and tables.

## References

[1] Siegel RL, Miller KD, Wagle NS, Jemal A (2023). Cancer statistics, 2023. CA Cancer J Clin.

[2] Morton D, Seymour M, Magill L, Handley K, Glasbey J, Glimelius B (2023). Preoperative Chemotherapy for Operable Colon Cancer: Mature Results of an International Randomized Controlled Trial. J Clin Oncol.

[3] Yue T, Li J, Zhu J, Zuo S, Wang X, Liu Y (2023). Hydrogen Sulfide Creates a Favorable Immune Microenvironment for Colon Cancer. Cancer Res.

[4] Ge T, Gu X, Jia R, Ge S, Chai P, Zhuang A (2022). Crosstalk between metabolic reprogramming and epigenetics in cancer: updates on mechanisms and therapeutic opportunities. Cancer Commun (Lond).

[5] Tan Y, Li J, Zhao G, Huang KC, Cardenas H, Wang Y (2022). Metabolic reprogramming from glycolysis to fatty acid uptake and beta-oxidation in platinum-resistant cancer cells. Nat Commun.

[6] Raggi C, Taddei ML, Rae C, Braconi C, Marra F (2022). Metabolic reprogramming in cholangiocarcinoma. J Hepatol.

[7] Lin J, Xia L, Oyang L, Liang J, Tan S, Wu N (2022). The POU2F1-ALDOA axis promotes the proliferation and chemoresistance of colon cancer cells by enhancing glycolysis and the pentose phosphate pathway activity. Oncogene.

[8] Zeng H, Pan T, Zhan M, Hailiwu R, Liu B, Yang H (2022). Suppression of PFKFB3-driven glycolysis restrains endothelial-to-mesenchymal transition and fibrotic response. Signal Transduct Target Ther.

[9] Guo D, Tong Y, Jiang X, Meng Y, Jiang H, Du L (2022). Aerobic glycolysis promotes tumor immune evasion by hexokinase2-mediated phosphorylation of IkappaBalpha. Cell Metab.

[10] Delaunay S, Pascual G, Feng B, Klann K, Behm M, Hotz-Wagenblatt A (2022). Mitochondrial RNA modifications shape metabolic plasticity in metastasis. Nature.

[11] Wan X, Li D, Lu J, Yan Y, He Z, Chen J (2023). The construction of garlic diallyl disulfide nano-emulsions and their effect on the physicochemical properties and heterocyclic aromatic amines formation in roasted pork. Food Chem.

[12] Tang H, Kong Y, Guo J, Tang Y, Xie X, Yang L (2013). Diallyl disulfide suppresses proliferation and induces apoptosis in human gastric cancer through Wnt-1 signaling pathway by up-regulation of miR-200b and miR-22. Cancer Lett.

[13] Zhao J, Huang WG, He J, Tan H, Liao QJ, Su Q (2006). Diallyl disulfide suppresses growth of HL-60 cell through increasing histone acetylation and p21WAF1 expression in vivo and in vitro. Acta Pharmacol Sin.

[14] Xia L, Lin J, Su J, Oyang L, Wang H, Tan S (2019). Diallyl disulfide inhibits colon cancer metastasis by suppressing Rac1-mediated epithelial-mesenchymal transition. Onco Targets Ther.

[15] Mitra S, Das R, Emran TB, Labib RK, Noor ET, Islam F (2022). Diallyl Disulfide: A Bioactive Garlic Compound with Anticancer Potential. Front Pharmacol.

[16] Malla R, Marni R, Chakraborty A, Kamal MA (2022). Diallyl disulfide and diallyl trisulfide in garlic as novel therapeutic agents to overcome drug resistance in breast cancer. J Pharm Anal.

[17] He H, Ma Y, Huang H, Huang C, Chen Z, Chen D (2021). A comprehensive understanding about the pharmacological effect of diallyl disulfide other than its anti-carcinogenic activities. Eur J Pharmacol.

[18] Colomer C, Margalef P, Villanueva A, Vert A, Pecharroman I, Sole L (2019). IKKalpha Kinase Regulates the DNA Damage Response and Drives Chemo-resistance in Cancer. Mol Cell.

[19] Roos WP, Kaina B (2006). DNA damage-induced cell death by apoptosis. Trends Mol Med.

[20] Wang HC, Yang JH, Hsieh SC, Sheen LY (2010). Allyl sulfides inhibit cell growth of skin cancer cells through induction of DNA damage mediated G2/M arrest and apoptosis. J Agric Food Chem.

[21] Murphy MP, Bayir H, Belousov V, Chang CJ, Davies KJA, Davies MJ (2022). Guidelines for measuring reactive oxygen species and oxidative damage in cells and in vivo. Nat Metab.

[22] Wikramanayake TC, Cheret J, Sevilla A, Birch-Machin M, Paus R (2022). Targeting mitochondria in dermatological therapy: beyond oxidative damage and skin aging. Expert Opin Ther Targets.

[23] Jin Z, Sinicrope FA (2022). Mismatch Repair-Deficient Colorectal Cancer: Building on Checkpoint Blockade. J Clin Oncol.

[24] Li Z, He Y, Li Y, Li J, Zhao H, Song G (2021). NeuroD1 promotes tumor cell proliferation and tumorigenesis by directly activating the pentose phosphate pathway in colorectal carcinoma. Oncogene.

[25] Cano-Cortina M, Alarcon L, Miranda J, Huber O, Gonzalez-Mariscal L (2022). Polyubiquitination and SUMOylation Sites Regulate the Stability of ZO-2 Protein and the Sealing of Tight Junctions. Cells.

[26] Cai C, Tang YD, Zhai J, Zheng C (2022). The RING finger protein family in health and disease. Signal Transduct Target Ther.

[27] Li JY, Zhao Y, Gong S, Wang MM, Liu X, He QM (2023). TRIM21 inhibits irradiation-induced mitochondrial DNA release and impairs antitumour immunity in nasopharyngeal carcinoma tumour models. Nat Commun.

[28] Cheng J, Huang Y, Zhang X, Yu Y, Wu S, Jiao J (2020). TRIM21 and PHLDA3 negatively regulate the crosstalk between the PI3K/AKT pathway and PPP metabolism. Nat Commun.

[29] Alrumaihi F, Khan MA, Babiker AY, Alsaweed M, Azam F, Allemailem KS (2022). The Effect of Liposomal Diallyl Disulfide and Oxaliplatin on Proliferation of Colorectal Cancer Cells: In Vitro and In Silico Analysis. Pharmaceutics.

[30] Filomeni G, Aquilano K, Rotilio G, Ciriolo MR (2005). Glutathione-related systems and modulation of extracellular signal-regulated kinases are involved in the resistance of AGS adenocarcinoma gastric cells to diallyl disulfide-induced apoptosis. Cancer Res.

[31] Hudlikar RR, Chou PJ, Kuo HD, Sargsyan D, Wu R, Kong AN (2023). Long term exposure of cigarette smoke condensate (CSC) mediates transcriptomic changes in normal human lung epithelial Beas-2b cells and protection by garlic compounds. Food Chem Toxicol.

[32] Pandey P, Khan F, Alshammari N, Saeed A, Aqil F, Saeed M (2023). Updates on the anticancer potential of garlic organosulfur compounds and their nanoformulations: Plant therapeutics in cancer management. Front Pharmacol.

[33] Kuo CH, Leu YL, Wang TH, Tseng WC, Feng CH, Wang SH (2019). A novel DNA repair inhibitor, diallyl disulfide (DADS), impairs DNA resection during DNA double-strand break repair by reducing Sae2 and Exo1 levels. DNA Repair (Amst).

[34] Jin Y, Schladetsch MA, Huang X, Balunas MJ, Wiemer AJ (2022). Stepping forward in antibody-drug conjugate development. Pharmacol Ther.

[35] Li Y, Liu X, Jiang D, Lin Y, Wang Y, Li Q (2016). Betulin induces reactive oxygen species-dependent apoptosis in human gastric cancer SGC7901 cells. Arch Pharm Res.

[36] Saxena S, Zou L (2022). Hallmarks of DNA replication stress. Mol Cell.

[37] Chen Y, Zhang H, Xu Z, Tang H, Geng A, Cai B (2019). A PARP1-BRG1-SIRT1 axis promotes HR repair by reducing nucleosome density at DNA damage sites. Nucleic Acids Res.

[38] Nedelcheva-Veleva MN, Krastev DB, Stoynov SS (2006). Coordination of DNA synthesis and replicative unwinding by the S-phase checkpoint pathways. Nucleic Acids Res.

[39] Wang HL, Chen Y, Wang YQ, Tao EW, Tan J, Liu QQ (2022). Sirtuin5 protects colorectal cancer from DNA damage by keeping nucleotide availability. Nat Commun.

[40] Li Q, Qin T, Bi Z, Hong H, Ding L, Chen J (2020). Rac1 activates non-oxidative pentose phosphate pathway to induce chemoresistance of breast cancer. Nat Commun.

[41] Milanese C, Bombardieri CR, Sepe S, Barnhoorn S, Payan-Gomez C, Caruso D (2019). DNA damage and transcription stress cause ATP-mediated redesign of metabolism and potentiation of anti-oxidant buffering. Nat Commun.

[42] Wang X, Zhang G, Dasgupta S, Niewold EL, Li C, Li Q (2022). ATF4 Protects the Heart From Failure by Antagonizing Oxidative Stress. Circ Res.

[43] Xie P, Peng Z, Chen Y, Li H, Du M, Tan Y (2021). Neddylation of PTEN regulates its nuclear import and promotes tumor development. Cell Res.

[44] Williams FP, Haubrich K, Perez-Borrajero C, Hennig J (2019). Emerging RNA-binding roles in the TRIM family of ubiquitin ligases. Biol Chem.

[45] Hatakeyama S (2011). TRIM proteins and cancer. Nat Rev Cancer.

[46] Zhu X, Xue J, Jiang X, Gong Y, Gao C, Cao T (2022). TRIM21 suppresses CHK1 activation by preferentially targeting CLASPIN for K63-linked ubiquitination. Nucleic Acids Res.

[47] Guha A, Nag S, Ray PS (2020). Negative feedback regulation by HuR controls TRIM21 expression and function in response to UV radiation. Sci Rep.

[48] Zhao Z, Wang Y, Yun D, Huang Q, Meng D, Li Q (2020). TRIM21 overexpression promotes tumor progression by regulating cell proliferation, cell migration and cell senescence in human glioma. Am J Cancer Res.

[49] Wang H, Zhou Y, Oyang L, Han Y, Xia L, Lin J (2019). LPLUNC1 stabilises PHB1 by counteracting TRIM21-mediated ubiquitination to inhibit NF-kappaB activity in nasopharyngeal carcinoma. Oncogene.

[50] Zhou G, Wu H, Lin J, Lin R, Feng B, Liu Z (2021). TRIM21 Is Decreased in Colitis-associated Cancer and Negatively Regulates Epithelial Carcinogenesis. Inflamm Bowel Dis.

[51] Si W, Zhou J, Zhao Y, Zheng J, Cui L (2020). SET7/9 promotes multiple malignant processes in breast cancer development via RUNX2 activation and is negatively regulated by TRIM21. Cell Death Dis.

[52] Ding Q, He D, He K, Zhang Q, Tang M, Dai J (2015). Downregulation of TRIM21 contributes to hepatocellular carcinoma carcinogenesis and indicates poor prognosis of cancers. Tumour Biol.

[53] Lee JH, Liu R, Li J, Zhang C, Wang Y, Cai Q (2017). Stabilization of phosphofructokinase 1 platelet isoform by AKT promotes tumorigenesis. Nat Commun.

[54] Li Z, Liu X, Zhang X, Zhang W, Gong M, Qin X (2022). TRIM21 aggravates cardiac injury after myocardial infarction by promoting M1 macrophage polarization. Front Immunol.

[55] Su J, Zhou Y, Pan Z, Shi L, Yang J, Liao A (2017). Downregulation of LIMK1-ADF/cofilin by DADS inhibits the migration and invasion of colon cancer. Sci Rep.

[56] Bellail AC, Jin HR, Lo HY, Jung SH, Hamdouchi C, Kim D (2021). Ubiquitination and degradation of SUMO1 by small-molecule degraders extends survival of mice with patient-derived tumors. Sci Transl Med.

[57] Li Q, Qin Y, Wei P, Lian P, Li Y, Xu Y (2016). Gas1 Inhibits Metastatic and Metabolic Phenotypes in Colorectal Carcinoma. Mol Cancer Res.

