# Supplementary material for: Diallyl disulfide induces DNA damage and growth inhibition in colorectal cancer cells by promoting POU2F1 ubiquitination
Source: Int J Biol Sci. 2024 Jan 21;20(4):1125–41. doi: 10.7150/ijbs.91206 (PMC10878159; doi:10.7150/ijbs.91206)
Supplement: Supplementary file 1 — Supplementary figures and tables. [file ijbsv20p1125s1.pdf]

Diallyl disulfide induces DNA damage and growth inhibition in colorectal cancer cells by promoting POU2F1 ubiquitination

Longzheng Xia[1*], Jinguan Lin[1*], Mingjing Peng[1*], [1#], Qianjin Liao[1,2,3#], Yujuan Zhou[1,2,3#]

1 Hunan Cancer Hospital and The Affiliated Cancer Hospital of Xiangya School of Medicine, Central South University, 283 Tongzipo Road, Changsha 410013, Hunan, China

2 Hengyang Medical School, University of South China, Hengyang, 421001, Hunan, China.

3 Public Service Platform of Tumor organoids Technology, 283 Tongzipo Road, Changsha, 410013, Hunan, China

*The authors Contribute equally to the work.

#Correspondence to: Yujuan Zhou or Qianjin Liao or Yingrui Shi, Hunan Key Laboratory of Translational Radiation Oncology, Hunan Cancer Hospital and The Affiliated Cancer Hospital of Xiangya School of Medicine, Central South University, 283 Tongzipo Road, Changsha 410013, Hunan, China. Tel: 86-731-88651681; Fax: 86-731-88651999; Email: yujany_zhou@163.com or march-on@126.com or shiyingrui@hnca.org.cn.

This content includes:

1) Supplementary Materials and Methods
2) Seven supplementary figures
3) Two supplementary tables

Supplementary Materials and Methods

Cell lines and culture

Human colorectal cancer (CRC) HCT116, SW620, SW480, and HT-29 cells were obtained from the Cancer Research Institute of Central South University and identified by short tandem repeat STR test. The SW620, SW480, and HT-29 cells were cultured in RPMI-1640 (Gibco, USA). The HCT116 and 293T cells were cultured in DMEM (Gibco, USA). All cells were cultured in medium supplemented with 10% fetal bovine serum (FBS, Zeta Life, France), 100 Units/ml of penicillin, and 100μg/ml of streptomycin (Gibco, USA) at 37 °C in 5% CO_2.

Immunofluorescent assays

Following treatment with DADS for two days, the expression levels of 53BP1 and γ-H_2AX in CRCCRC cells were characterized by immunofluorescence using anti-53BP1 (1:100 dilution) and anti-H_2AX (1: 50 dilution), as well as optimal fluorescent secondary antibodies in a confocal microscope (LSM510 META, ZEISS, Germany).

RNA extraction and quantitative real-time PCR (RT-qPCR)

Total cellular RNA was extracted using TRIzol reagent (Invitrogen, 15596-018), and reversely transcribed into cDNA using Revert Aid First Strand cDNA Synthesis Kit (Thermo scientific, Massachusetts, USA), according to the manufacturer's instruction. The relative levels of targeted gene mRNA transcripts to the control α-tubulin were determined by RT-qPCR using a Fast Start Essential DNA Green Master kit (Lifescience, Roche, Mannheim, Germany) and specific primers (**Supplementary Table S1**) in the Roche Light Cycler® 96 Instrument (Lifescience). The PCR reactions were performed in triplicate at 95°C for 5 min and subjected to 40 cycles of 95°C (30 s), 60°C (10 s), and 72°C (1 min). The data were analyzed by $2^{-\Delta\Delta Ct}$.

Western blot and co-immunoprecipitation

The relative levels of targeting proteins were determined by Western blot assays. Individual cell lysates (30 μg/lane) were separated by sodium dodecyl sulfate-polyacrylamide gel electrophoresis (SDS-PAGE) on 10% gels, and transferred onto PVDF membranes. After being blocked with 5% fat-free dry milk in TBST, the membranes were incubated overnight at 4°C

with primary antibodies (**Supplementary Tables S2**). The bound antibodies were detected by horseradish peroxidase (HRP)-conjugated second antibodies, and visualized using Pierce™ ECL Western Blotting Substrate (Thermo Scientific). The levels of targeting proteins were quantified by densitometric scanning using ImageJ software. For Co-IP, proteins were immunoprecipitated by anti-POU2F1 or TRIM21 antibody and microbeads. The bound proteins were analyzed by Western blot assays using antibodies against POU2F1 and TRIM21.

Immunohistochemistry (IHC)

Individual paraffin-embedded tissues were dewaxed and rehydrated. The tissue sections (4 μm) were stained with primary antibodies overnight at 4 °C and the bound antibodies were detected with HRP-conjugated second antibodies, followed by visualizing with DAB, according to the immunohistochemical kit protocols (Cwbiotech, Beijing, China). The primary antibodies included anti-Bax, anti-Bcl-2, anti-cleave capase3, anti-POU2F1, anti-HK2, anti-G6PD and anti-RPIA. The samples were scored by two pathologists in a blinded manner, according to the staining intensity and percent of positive cells, as described previously (1) staining intensity: 0, no observed cell staining; 1, cells with weak staining: 2, cells with moderate staining; 3 cells with strong staining; (2) percent of positive cells: 0, no positive cells; 1, less than 25% of positive cells; 2, between 25% and 50% positive cells; 3, positive cells over 50%. Next, the IHC score of individual sections was obtained by multiplying the intensity score and positive cell frequency score, leading to a maximum score of 9. A section with IHC score of < 4 was designated as low expressions. A total of three sections from individual specimen were analyzed.

Supplementary Figures legends

Figure S1

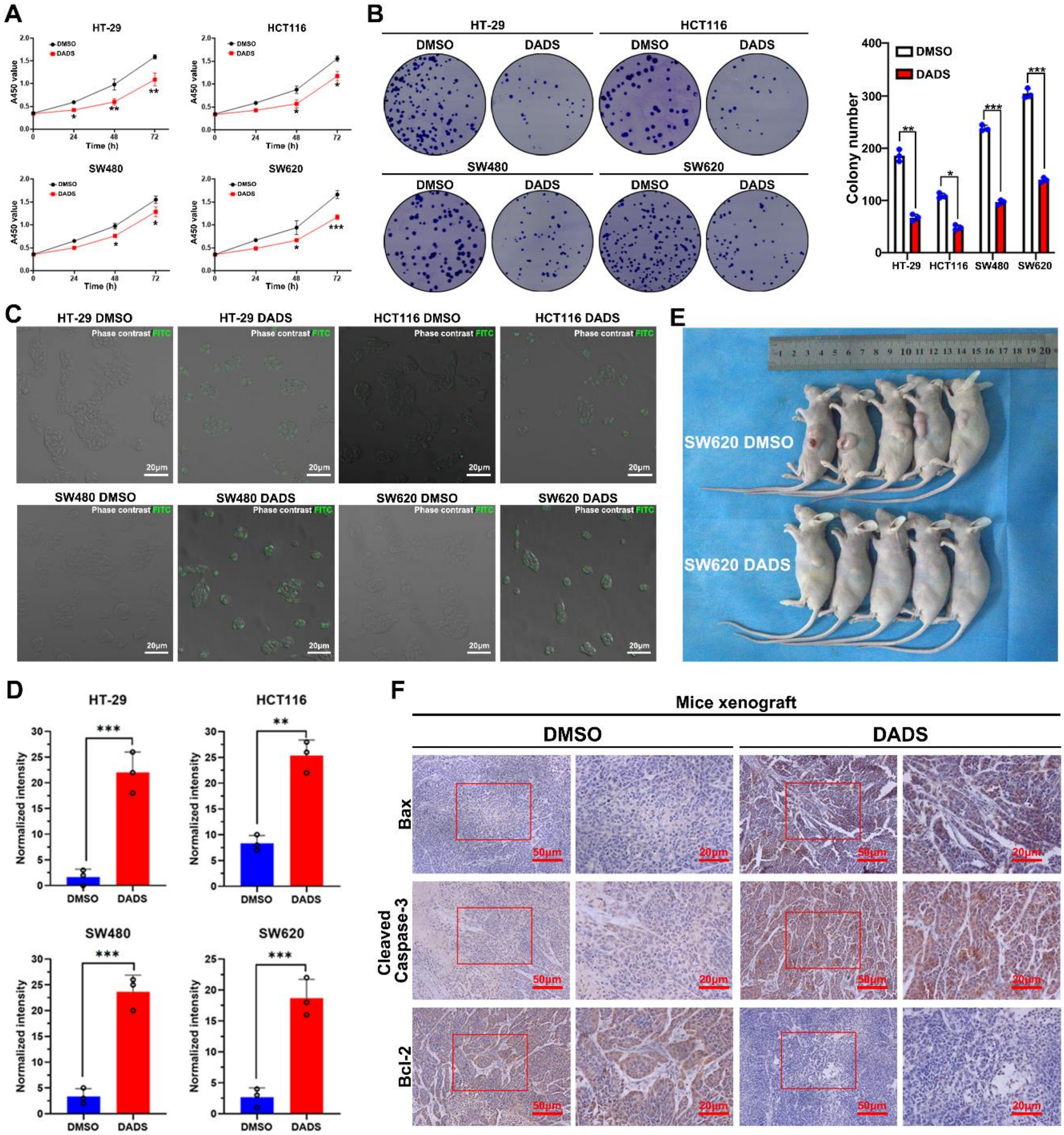

Figure S1. DADS induces the growth inhibition and apoptosis of CRC cells.

(**A-B**) DADS inhibited the proliferation and clonogenicity of HT-29, HCT116, SW620 and SW620 cells, determined by CCK8 assays and colony formation assays, respectively. (**C-D**) Cell apoptosis was examined by the TUNEL assay. Representative images of cell apoptosis are shown. Scale bars, 20 μm. The number of TUNEL-positive nuclei per field was counted in five randomly micrographs for each sample. (**E**) Photoimages of mice bearing SW620/DMSO, or SW620/DADS tumors. (**F**) Immunohistochemistry images of the Bax, cleave caspase 3 and

Bcl-2 expression (magnification x200, scale bars 50 μm, magnification x400, scale bars 20 μm). Data are representative images or expressed as the mean ± SD of each group of samples from three separate experiments. *$P<0.05$, **$P<0.01$, ***$P<0.001$.

Figure S2

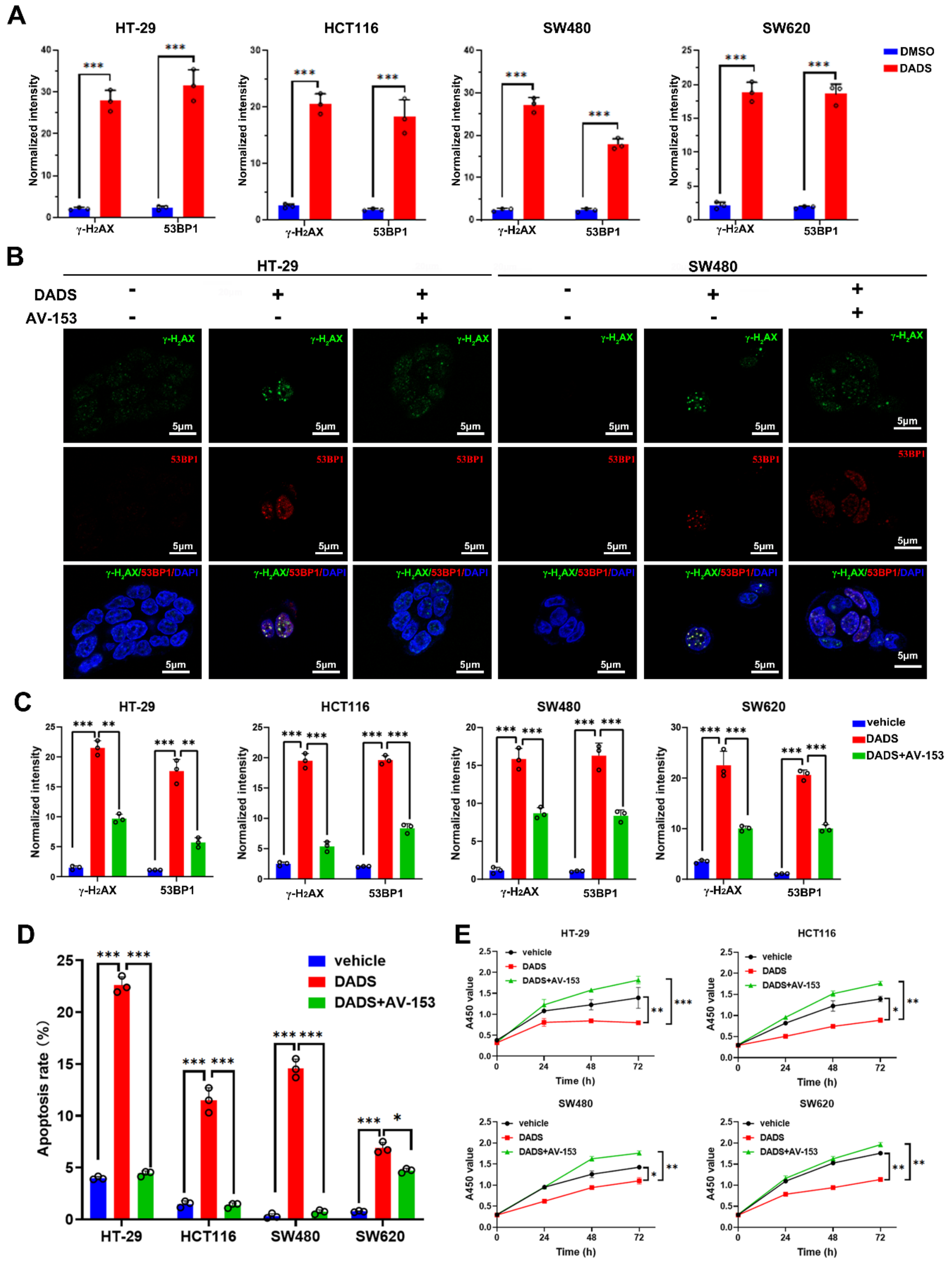

Figure S2. DADS enhances DNA damage in CRC cells.

(**A**) Normalized fluorescence intensity of γ-H_2AX and 53BP1 in cells stimulated as in indicated cells following treatment with vehicle or DADS. (**B-C**) Immunofluorescent analysis of γ-H_2AX

and 53BP1 expression in the indicated cells following treatment with vehicle or DADS alone or combination with10 mM AV-153 (magnification x 1000, scale bars 5 μm). (**D-E**) Flow cytometry analysis of the apoptosis rate (**D**) and CCK-8 analysis of the viability (**E**) of the indicated cells following treatment with vehicle or DADS alone or combination with10 mM AV-153. Data are representative images or expressed as the mean ± SD of each group of samples from three separate experiments. *P<0.05, **P<0.01, ***P<0.001.

Figure S3

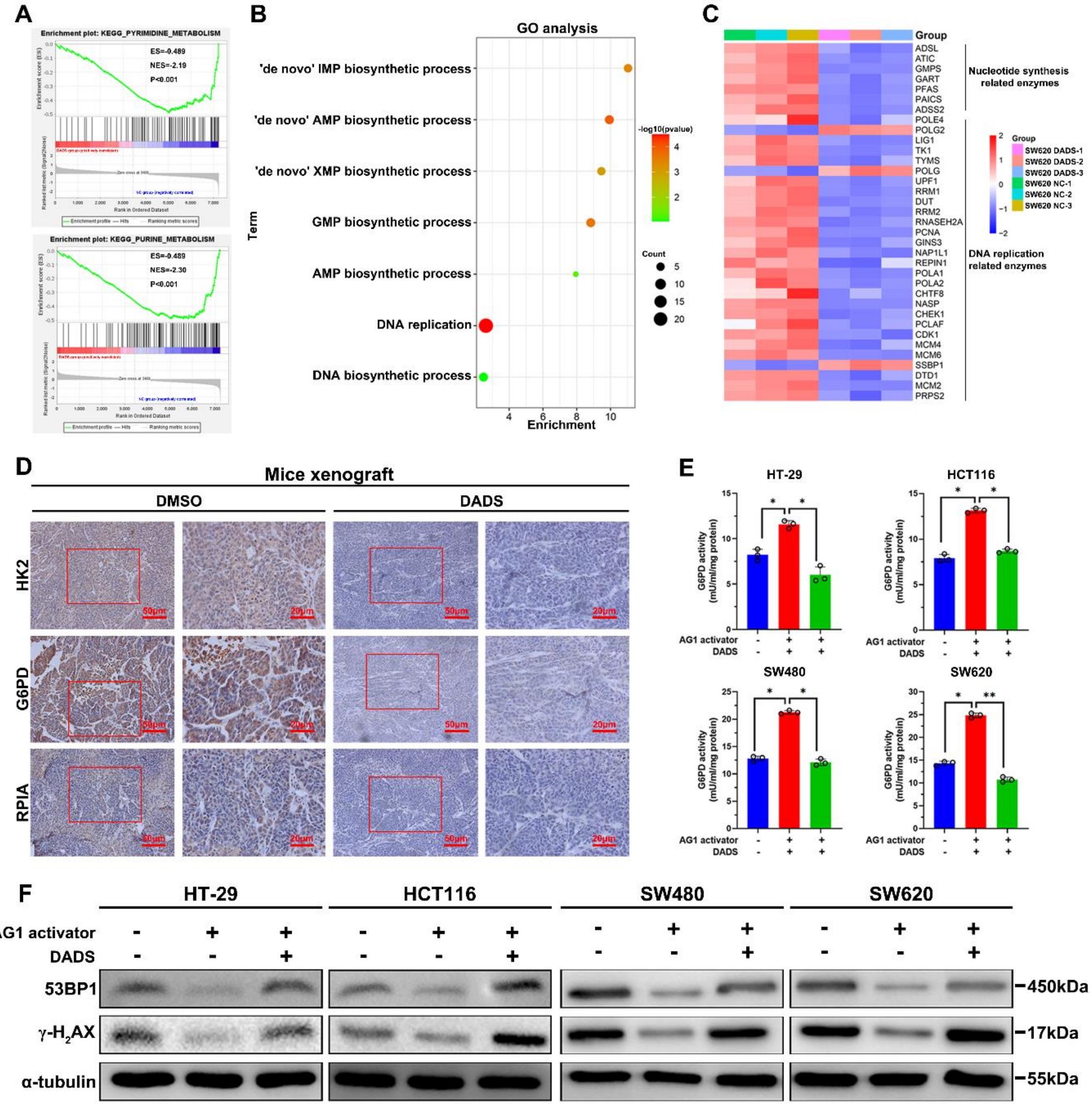

Figure S3. DADS suppresses the PPP activity in CRC cells.

(**A**) GSEA analysis predicted the purine and pyrimidine metabolism were enriched in the control group. (**B-C**) GO and Heatmap analysis indicated that DADS treatment altered the nucleotide synthesis in CRC cells. (**D**) Immunohistochemistry images of the HK2, G6PD and RPIA expression (magnification x 200, scale bars 50 μm, magnification x 400, scale bars 20 μm). (**E**) DADS treatment mitigated or abrogated the AG activator 1-enhanced G6PD activities in CRC cells. (**F**) DADS rescued the AG activator 1-decreased γ-H_2AX and 53BP1 expression in CRC cells. Data are representative images or expressed as the mean ± SD of each group of samples from three separate experiments. *P<0.05, **P<0.01, ***P<0.001.

Figure S4

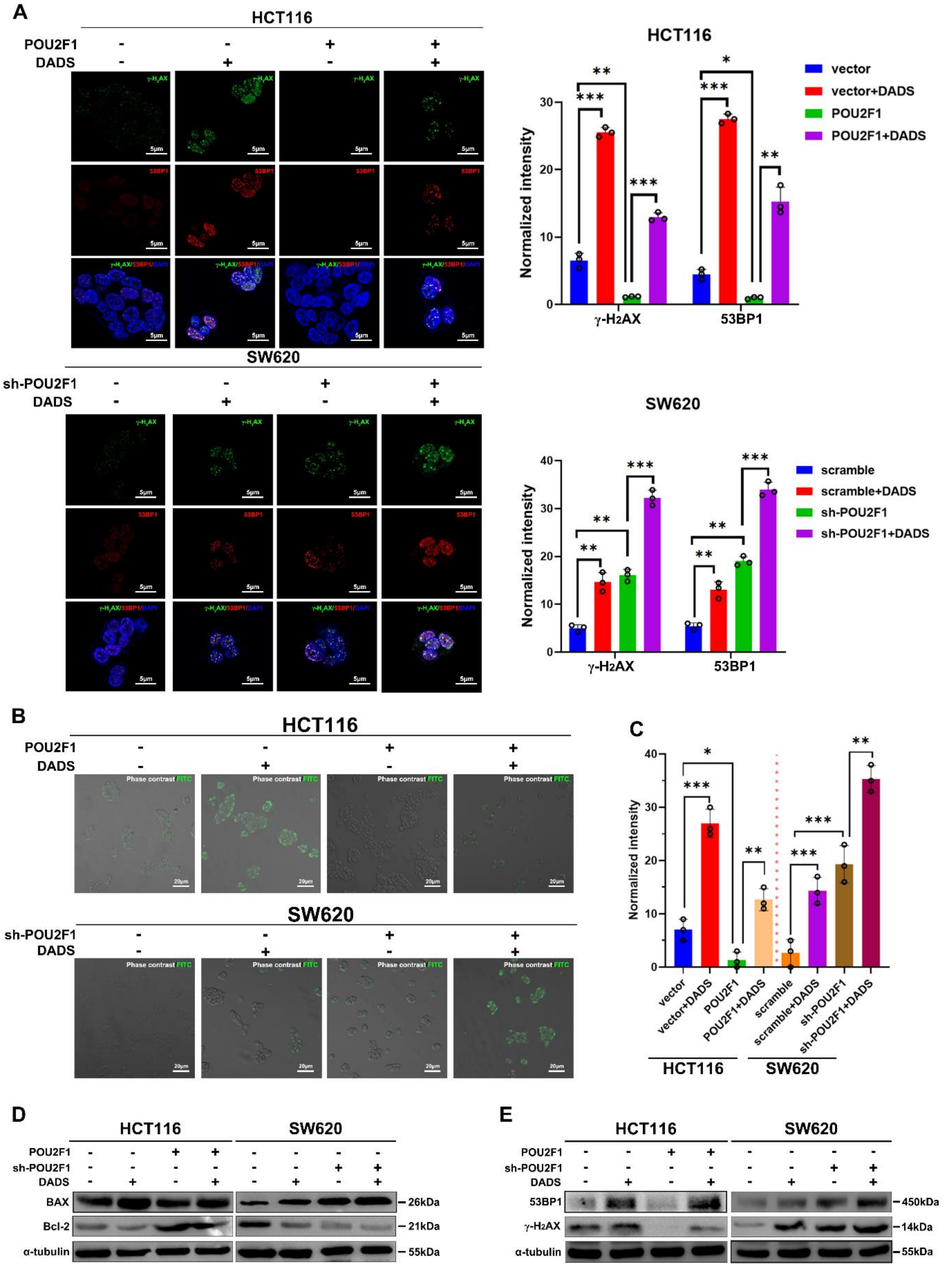

Figure S4. DADS attenuates the inhibition of POU2F1 on DNA damage in CRC cells.

(**A**) Immunofluorescent analysis of γ-H_2AX and 53BP1 expression in the indicated cells

following treatment with DADS. (**B-C**) TUNEL assay of the frequency of apoptotic CRC cells. (**D-E**) Western blot analyses of the relative levels of Bax, Bcl-2 γ-H_2AX and 53BP1 expression in the indicated cells following treatment with DADS. Data are representative images or expressed as the mean ± SD of each group of samples from three separate experiments. *$P<0.05$, **$P<0.01$, ***$P<0.001$.

Figure S5

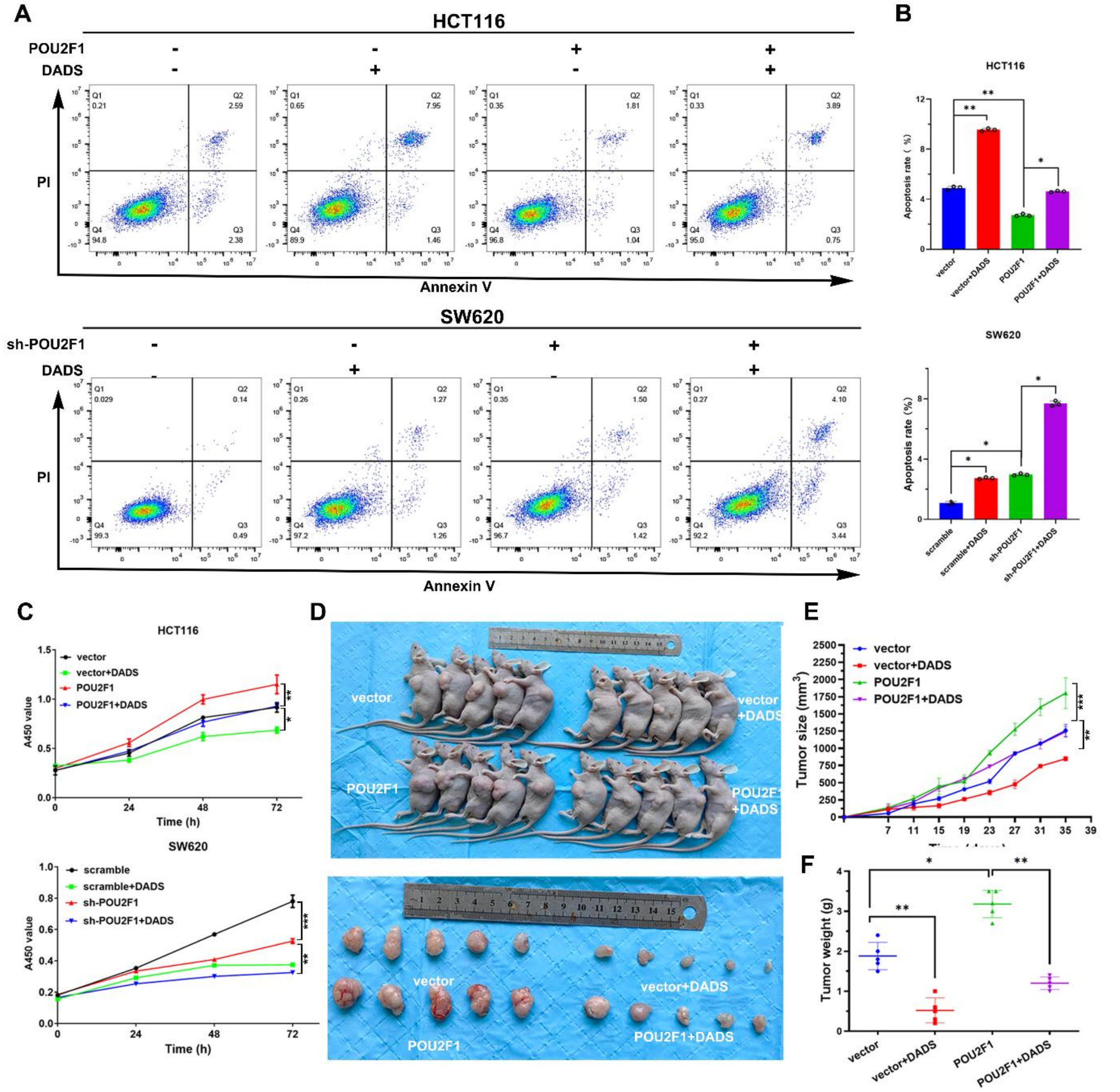

Figure S5. DADS attenuates the promoting effect of POU2F1 on the growth of CRC cells. (**A-C**) The cell apoptosis and proliferation were determined by flow cytometry and CCK8 assays in the indicated cells with DADS treatment, respectively. (**D-E**) DADS attenuated the growth of POU2F1-overexpressing HCT116 tumors in mice. Individual BALB/c nude mice were injected subcutaneously with 5×10^6 cells indicated. When the tumors reached at 200 mm^3, the mice were injected intraperitoneally with DADS (100 mg/kg) every other day up to 4 weeks. The dynamic growth of tumors was measured longitudinally (n=5 per group). At the end of observation, their tumors were dissected, photoimaged, and weighed. Data are representative images or expressed as the mean ± SD of each group of samples from three separate experiments. Ns: no significance, *P<0.05, **P<0.01, ***P<0.001.

Figure S6

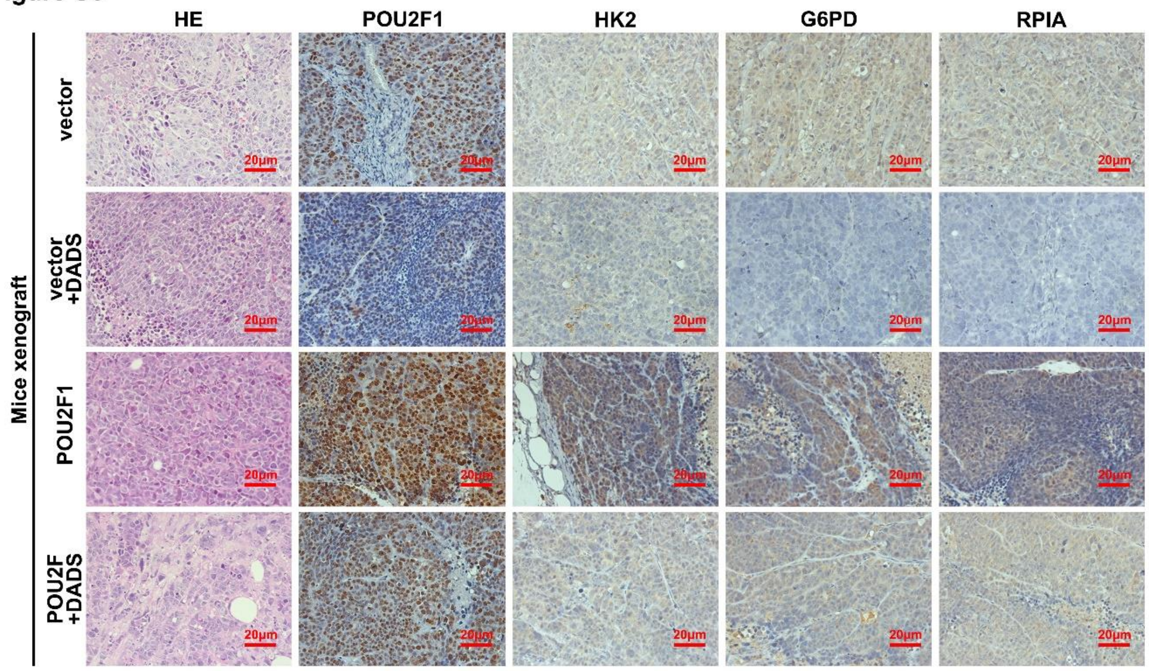

Figure S6. Immunohistochemistry analysis of POU2F1, HK2, G6PD and RPIA expression in SW620 xenograft tumors.

Data shown are representative images (magnification x 400, scale bars 20 μm).

Figure S7

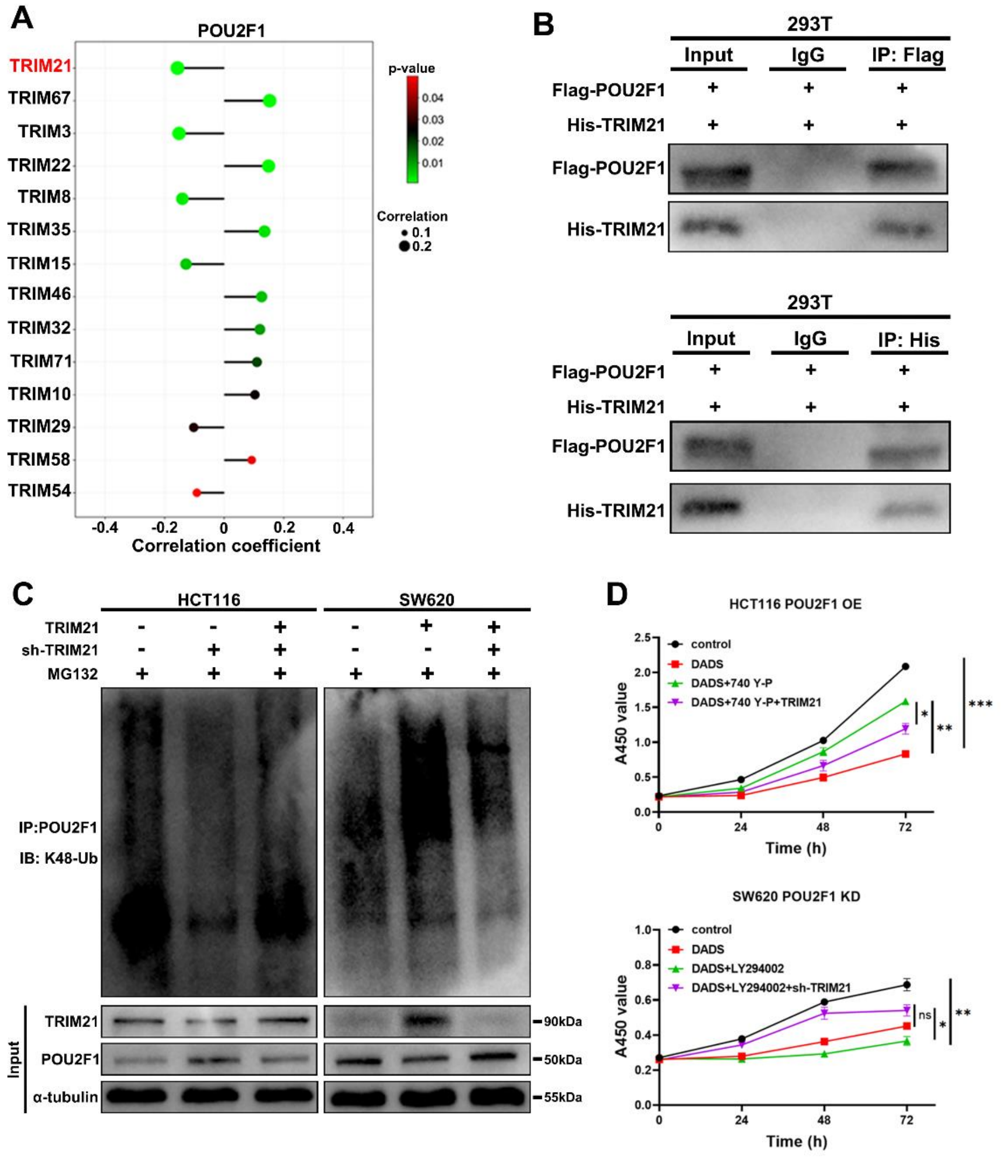

Figure S7. TRIM21 is a mediator of POU2F1 ubiquitination and inhibits the proliferation of CRC cells.

(**A**) Visualization of correlation between POU2F1 and TRIM family members in TCGA-COAD cohort. (**B**) Co-IP revealed that TRIM21 directly interacted with POU2F1 in 293T cells. 293T cells were transfected with the plasmids for Flag-POU2F1 and His-TRIM21 expression and the contained POU2F1 and TRIM21 were immunoprecipitated using an anti-Flag antibody and anti-His, respectively, followed by Western blot analysis. (**C**) Enforced TRIM21 expression enhances the POU2F1 ubiquitination in TRIM21-silencing HCT116 cells. (**D**) The cell proliferation was determined by CCK8 assays in the indicated cells. Data are representative images or expressed as the mean ± SD of each group of samples from three separate

experiments. Ns: no significance, *P<0.05, **P<0.01, ***P<0.001.

Supplementary Tables

Table S1. The sequences of primers for RT-qPCR

**Genes**	**Refseq No.**	**Forward (5'-3')**	**Reverse (5'-3')**
POU2F1	NM_001198783.2	GATCTGCCTGGTGAATGCTG	ATCGCCGCAAAACATCTCTC
TRIM21	NM_003141.4	AGGTCTCCACACTGCTGTTT	GTTGGCTAGCTGTCGATTGG
α-tubulin	NM_006009.4	TACGGAAAGAAGTCCAAGC	CTGAGGGAAGCAGTGATG

Table S2. Antibodies for Western blotting, immunofluorescence, and immunohistochemistry

Antibody	Catalog number	Company	Experiment	Dilution
anti-POU2F1	#8157	CST	Western Blotting	1:500
			Immunofluorescence	1:100
	ab178869	Abcam	Immunohistochemistry	1:50
anti-Bax	ab32503	Abcam	Western Blotting	1:500
			Immunohistochemistry	1:50
anti-Bcl-2	ab182858	Abcam	Western Blotting	1:500
			Immunohistochemistry	1:50
anti-HK2	ab209847	Abcam	Western Blotting	1:500
			Immunohistochemistry	1:100
anti-G6PD	ab133525	Abcam	Western Blotting	1:500
			Immunohistochemistry	1:50
anti-RPIA	13010-1-AP	Proteintech	Western Blotting	1:400
			Immunohistochemistry	1:100
anti-cleave caspase 3	ab32042	Abcam	Immunohistochemistry	1:100
anti-γ-H_2AX	ab11175	Abcam	Western Blotting	1:500
			Immunofluorescence	1:100
anti-53BP1	ab175933	Abcam	Western Blotting	1:500
			Immunofluorescence	1:100
anti-α-tubulin	AF0001	Beyotime Biotechnology	Western Blotting	1:1000
anti-His	ab200537	Abcam	Western Blotting	1:500
			Immunoprecipitation	1:50
anti-Flag	MA1-91878	Invitrogen	Western Blotting	1:500

			Immunoprecipitation	1:50
anti-K48 Ub	ab140601	Abcam	Western Blotting	1:500
anti-K63 Ub	ab179434	Abcam	Western Blotting	1:500
anti-Ubiquitin	ab134953	Abcam	Western Blotting	1:500
anti-HA	ab236632	Abcam	Western Blotting	1:500
			Immunoprecipitation	1:50
anti-α-tubulin	ab7291	Abcam	Western Blotting	1:500
